# Within patient genetic diversity of *bla*_KPC_ harboring *Klebsiellapneumoniae* in a Colombian hospital and identification of a new NTE_KPC_ platform

**DOI:** 10.1038/s41598-021-00887-2

**Published:** 2021-11-01

**Authors:** Deisy Abril, Erika Vergara, Diana Palacios, Aura Lucía Leal, Ricaurte Alejandro Marquez-Ortiz, Johana Madroñero, Zayda Lorena Corredor Rozo, Zandra De La Rosa, Carlos A. Nieto, Natasha Vanegas, Jorge A. Cortés, Javier Escobar-Perez

**Affiliations:** 1 Bacterial Molecular Genetics Laboratory, Universidad El Bosque, Carrera 9 #131A-02, Bogota, Colombia; 2 Facultad de Medicina y Grupo de Investigación en Enfermedades Infecciosas, Universidad Nacional de Colombia, Bogotá, Colombia; 3 Departamento de Patología y Laboratorios, Fundación Santa Fe de Bogotá, Bogotá, Colombia; 4 Facultad de Medicina, Departamento de Patología, Universidad Nacional de Colombia, Bogotá, Colombia; 5 The i3 Institute, Faculty of Science, University of Technology, Sydney, Australia

**Keywords:** Genetics, Microbiology, Molecular biology

## Abstract

Resistance to carbapenems in *Klebsiella* *pneumoniae* has been mostly related with the worldwide dissemination of KPC, largely due to the pandemic clones belonging to the complex clonal (CC) 258. To unravel *bla*_KPC_ post-endemic clinical impact, here we describe clinical characteristics of 68 patients from a high complexity hospital, and the molecular and genetic characteristics of their 139 *bla*_KPC_—*K.* *pneumoniae* (KPC-*Kp*) isolates. Of the 26 patients that presented relapses or reinfections, 16 had changes in the resistance profiles of the isolates recovered from the recurrent episodes. In respect to the genetic diversity of KPC-*Kp* isolates, PFGE revealed 45 different clonal complexes (CC). MLST for 12 representative clones showed ST258 was present in the most frequent CC (23.0%), however, remaining 11 representative clones belonged to non-CC258 STs (77.0%). Interestingly, 16 patients presented within-patient genetic diversity of KPC-*Kp* clones. In one of these, three unrelated KPC-*Kp* clones (ST258, ST504, and ST846) and a *bla*_KPC_—*K.* *variicola* isolate (ST182) were identified. For this patient, complete genome sequence of one representative isolate of each clone was determined. In *K.* *pneumoniae* isolates *bla*_KPC_ was mobilized by two Tn*3*-like unrelated platforms: Tn*4401b* (ST258) and Tn*6454* (ST504 and ST846)*,* a new NTE_KPC-_IIe transposon for first time characterized also determined in the *K.* *variicola* isolate of this study. Genome analysis showed these transposons were harbored in different unrelated but previously reported plasmids and in the chromosome of a *K.* *pneumoniae* (for Tn*4401b*). In conclusion, in the *bla*_KPC_ post-endemic dissemination in Colombia, different KPC-*Kp* clones (mostly non-CC258) have emerged due to integration of the single *bla*_KPC_ gene in new genetic platforms. This work also shows the intra-patient resistant and genetic diversity of KPC-*Kp* isolates. This circulation dynamic could impact the effectiveness of long-term treatments.

## Introduction

The carbapenem resistance in *K.* *pneumoniae* is a major public health problem because carbapenems are broad spectrum β-lactams with efficacy in severe infections caused by extended spectrum β-lactamase (ESBL) producing strains^1^. Carbapenem resistance in this species has been mainly attributed to the production of carbapenemases, principally, *K.* *pneumoniae* carbapenemase (KPC) and decreased membrane permeability in the cell wall^2^. Since the first report of KPC in a North Carolina hospital almost 30 years ago, KPC-producing *K.* *pneumoniae* (KPC-*Kp*) isolates have been endemically spread throughout the world, causing a wide range of severe infections^3,4^. To date, a great number of *bla*_KPC_ variants has been reported in different *Enterobacteriaceae* species and Nonfermenting Gram-negative Bacilli, however, the *bla*_KPC-2_ and *bla*_KPC-3_ variants still are the most frequent^5^. The wide dissemination of the *bla*_KPC_ gene has been attributed to plasmids harboring isoforms (a-i) of the Tn*4401* transposon and recently to *bla*_KPC_-bearing non-Tn*4401* elements (NTE_KPC_)^6–13^.

The KPC-*Kp* global expansion has been principally associated to pandemic clones of the clonal complex 258 (CC258), formed by ST258 and its single locus variants (SLVs) ST11, ST340, ST512, and more than 100 ST’s that have been described^14,15^. Most genomic diversity analyses of KPC-*Kp* compare isolates from different patients but only a few studies have examined variation within the same patient (intra-patient variation). One study of KPC-*Kp* isolates from a French patient over 4.5 years showed the genetic adaptation of the ST258 clone to antibiotic treatments during colonization^16^. In other case an Italian patient with a KPC-*Kp* isolate from a rectal swab, remained colonized during all hospital stay and exhibited changes in the phenotypic resistance due to mutations in the *bla*_KPC-3_ gene, showing the emergence of subpopulations with different resistant profiles as a response to antimicrobial treatment^17^.

In Colombia, few studies have analyzed the genetic relationship of KPC-*Kp* isolates, and these results indicated that the initial dissemination of *bla*_KPC_ in this species was ruled by non-CC258 that acquired this gene in a Tn*4401* probably by horizontal transfer. Later on, some CC258 lineages have been introduced, nonetheless *bla*_KPC_ endemic dissemination has been characterized by spreading of CC258 and non-CC258 lineages^18,19^. In line with that observation, after the settlement of *bla*_KPC_ (post-endemic era), resistance profiles of KPC-*Kp* seem to show a diversity in the population, suggesting the involvement of different genetic backgrounds in the *bla*_KPC_ dissemination^20^. Moreover, information describing intra-patient variations in patients with recurrent KPC-*Kp* infections support the possibility of harboring distinct resistant subclones with high genetic diversity^16,17^. Since 2013, we have observed high inter- and intra-patient variation in phenotypic resistance profiles of isolates from patients admitted to a high complexity institution in Colombia. That motivated us to study the post-endemic intra and inter-patient genetic variability of KPC-*Kp* isolates from a cohort of patients from this institution, to understand the role of CC258 and non-CC258 in the *bla*_KPC_ dissemination. Here, we show a complex scenery where both CC258 related and non-related can affect a single patient. Furthermore, our results showed that in the post-endemic era, *bla*_KPC_ as a single unit has disseminated toward novel (NTE_KPC_) fully functional genetic platforms in non-CC258 clones.

## Methods

### Clinical analysis of the patients

Medical records for 68 hospitalized patients with KPC-*Kp* isolates were reviewed in a retrospective and descriptive study between 2014 and 2016 in a high complexity hospital in Bogota, Colombia, within the framework of an inter-institutional project, approved by the institutional ethics committee (CCET-2726-2015). Demographic variables, medical history, type of hospitalization (medical or surgical cause), degree of comorbidity (Charlson index), clinical diagnosis, use of antibiotics in the last year, requirement for management in an intensive care unit, antibiotic therapy (until death or discharge) and final outcome (death or discharge) were included. Each clinical bacterial isolate (either infection or colonization, according to medical criterium) was defined as an event, and in those patients, who presented more than one event, all isolates were analyzed. First events presented after 48 h of the hospital admission were classified as hospital acquired. If the first event happened in the first 48 h, it was considered from community, unless the patient had any health care related antecedent in the previous 6 months; those cases were classified as healthcare associated. Subsequent events were classified according to the source, diagnosis, and clinical significance.

### Bacterial Isolates and susceptibility profile

In the 68 patients, 139 KPC-*Kp* isolates were recovered. Susceptibility profiles of the isolates to ampicillin, ampicillin/sulbactam, piperacillin/tazobactam, cephalothin, cefoxitin, ceftazidime, ceftriaxone, cefepime, meropenem, ertapenem, doripenem, amikacin, gentamicin, ciprofloxacin, trimethoprim/sulfamethoxazol and colistin were determined by the automated system VITEK^®^ 2 using the breakpoints defined by the CLSI, according to M100-S25 2017 update.

### PCR detection of resistance genes and genetic relation establishment

The DNA of each isolate was extracted using the Wizard^®^ Genomic DNA Purification kit (Promega) following manufacturer's instructions. *K.* *pneumoniae* isolates were confirmed by the amplification of the *khe* gene, and possible contamination with other species was ruled out by amplifying the genes *pehX* (*K.* *oxytoca*), *uidA* (*E.* *coli*), *ehe* (*Enterobacter* spp*.*), *wosA* (*P.* *mirabilis*) by multiplex PCR (table S1). Presence of relevant β-lactamases genes in all isolates was assessed by multiplex PCR (*bla*_TEM_*,* *bla*_CTX-M,_ *bla*_GES_, *bla*_IMP_, *bla*_VIM_, *bla*_KPC_ and *bla*_NDM_) using previously described protocols^21,22^. The genetic relatedness between isolates was determined by genome macro-restriction using the *Xba*I enzyme (Thermo Scientific) and PFGE separation according to the protocol reported by Herschleb et al.^23^. PFGE pulsotypes were grouped in the same clonal complex (CC) when they presented no more than three different bands. Sequence type (ST) to some representative isolates of the most frequent clonal complexes was determined using the protocol reported by Diancourt et al.^24^.

### Whole genome sequencing

Three KPC-*Kp* isolates representing three clones of the patient with the highest number of events and an additional *K.* *variicola* isolate (from the same patient) were selected for comparative genomic analysis. From an exponential growth culture, total DNA was extracted following the protocol of the commercial UltraClean^®^ Microbial DNA Isolation Kit (QIAGEN N.V). For each bacterial DNA, a 20 kb enriched SMRTbellTM library was prepared, and libraries were sequenced using the PacBio RS II platform with the P6-C4 chemistry and one SMRT cell per sample. The reads obtained were assembled de novo using the HGAP program in the SMRT Analysis v2.3. The contigs obtained were compared with each other for the presence of repeated ends, suggestive of complete circular structures, through Artemis Comparison Tool (ACT) and dot plot analysis with the GEPARD 1.30^25,26^. For quality control sequences of the contigs were evaluated by mapping of the reads using BWA-MEM^27^. Preliminary annotation of open reading frames was done in Prokka v1.13^28^ and manually curated in the regions of interest. Complete sequences were circularized as previously described^29^. Randomly generated starting positions in complete replicons were adjusted using Circlator according to the *dnaA* (chromosome) or *rep* genes (plasmids), when possible^30^. For plasmids the pMLST were determined using PlasmidFinder, pMLST and Plasmid MLST^31^. Antibiotic resistance genes were identified using ResFinder^32^ and CARD^33^. Pairwise comparisons were plotted using Easyfig^34^. The putative promoter regions between *bla*_KPC_ and *tnpR* genes were predicted by Bacterial Promoter Prediction Program (BPROM [Softberry]) with an LDF threshold of 0.2.

### The Tn*3* family analysis

The protein sequences of the transposases and S-recombinases of 19 Tn*3*-family mobile elements, including those reported here (available at ISFinder^35^ and The Transposon Registry^36^) belonging of seven TnpA subgroups proposed by Nicolas et al.^37^ were aligned with MAFFT (table S2)^38^. The alignment of the transposases in ClustalW format was visualized in Jalview 2.11.1.1^39^ to identify the DDE motif. The phylogenetic analysis of the sequences was carried out using the neighbor-joining (NJ) method running 1000 bootstrap replicates. Finally, the consensus tree was plotted using Dendroscope^40^. Branch lengths were expressed in changes/nucleotide position (scale bar).

### Ethics committee

This study was approved by Corporate Research Ethics Committee of Fundación Santa Fe de Bogotá (CCET-Z726-20L5) and the Institutional Ethics Committee of Universidad El Bosque (UB-441-2017) and declared without risk by Colombian Resolution 8430 of 1993. The patients signed an informed consent to participate; for underage patients (below 18 age), the consent was signed from a parent and/or legal guardian. All experiments were performed in accordance with Declaration of Helsinki and Colombian Resolutions (8430 of 1993 and 2378 of 2008).

### Accession numbers of the KPC-*Kp* genomes

The chromosome and plasmids sequences of the four *Klebsiella* isolates sequenced in this study have been published in the Nucleotide database, with the following accession numbers. For KPC-*Kp* isolates: 33Kpn9 (CP064296), p33Kpn9-KPC (CP064297) and p33Kpn9-2 (CP064298); 33Kpn12 (CP062792) p33Kpn12-1 (CP062793) and p33Kpn12-KPC (CP062794); 33Kpn22 (CP069046), p33Kpn22-1 (CP069047), p33Kpn22-2 (CP069048), p33Kpn22-3 (CP069049), p33Kpn22-KPC (CP069050) and p33Kpn22-5 (CP069051). For KPC-*Kv* isolate 33Kva16 (JAGTWT010000001- JAGTWT010000007), p33Kva16-1 (JAGTWT010000008) and p33Kva16-KPC (JAGTWT010000009).

## Results

### Clinical and epidemiological characteristic of the patients

In this study, 139 KPC-*Kp* isolates were recovered from 68 patients treated from February 2014 to December 2016. The proportion of men and women was similar (35/33) with a mean age of 56 years (ranging from 14 to 94). The population had an average Charlson index of 4.6 (range 0–11). The most frequent comorbidities were immunosuppression (no HIV), diabetes, cirrhosis, and cancer with 28 (41.2%), 19 (27.9%), 19 (27.9%), and 19 (27.9%) cases, respectively. Sixty-five patients (95.6%) received some type of antibiotic in the last year before the first event, including the carbapenem treatment that had been given in 44.9% of the patients (Tables 1, 2).

**Table 1:** Clinical characteristics of the 69 hospitalized patients with KPC-*Kp* isolates during 2014–2016.

Variables	n (%)	Median (IQ range)
**Gender**		
Male	35 (51.5)	
Female	33 (48.5)	
**Age** **(years)**		
< 20	5 (7.3)	56 (42–71)
20–39	9 (13.2)	
40–59	23 (33.8)	
60–79	20 (29.4)	
≥ 80	11 (16.2)	
**Comorbidities**		
Immunosuppression*	28 (41.2)	
Cancer	19 (27.9)	
Solid organ	15 (22.0)	
Haematologic	4 (5.9)	
Cirrhosis	19 (27.9)	
DM	19 (27.9)	
CKD	15 (22.0)	
COPD	6 (8.8)	
Charlson index		4.6 (0–11)
Antibiotic in last year	65 (95.6)	
Penicillins and inhibitors	42 (61.8)	
1st or 2nd generation cephalosporins	3 (4.4)	
3rd generation cephalosporins	6 (8.8)	
4th generation cephalosporins	13 (19.1)	
Carbapenem	30 (44.1)	
Quinolones	10 (14.7)	
Polymyxin	8 (11.8)	
Tigecycline	4 (5.9)	
Aminoglycoside	3 (4.4)	
Fosfomycin	2 (2.9)	
**Hospitalization** **cause**		
Surgical	35 (51.4)	
Medical	33 (48.5)	
ICU admission	45 (66.2)	
APACHE		18 (5–26)
SOFA		6 (1 A 18)
Hospital stay		29 (14–65) days
Death	12 (17.6)	

*DM* diabetes mellitus, *CKD* chronic kidney disease, *COPD* chronic obstructive pulmonary disease.

*Patients with immunosuppression except HIV.

**Table 2:** Clinical and microbiological information of the events in which KPC-*Kp* isolates were identified.

Variable	n	%
**Antibiotic** **use** **prior** **to** **the** **event**	136	97.8
Penicillins and inhibitors	103	74.1
1st or 2nd generation cephalosporins	6	4.3
3rd generation cephalosporins	18	12.9
4th generation cephalosporins	30	21.6
Carbapenem	97	69.8
Fosfomycin	33	23.7
Polymyxin	28	20.1
Aminoglycoside	20	14.4
Tigecycline	20	14.4
Quinolones	19	13.7
**Infectious** **diagnosis**		
Clinically significant	86	61.9
Urinary tract infection	42	30.2
Bacteremia	17	12.2
Gastrointestinal focus	8	5.7
Associated with catheter	6	4.3
Primary bacteremia	1	0.7
Febrile neutropenia	1	0.7
Urinary focus	1	0.7
Peritonitis	13	9.3
Surgical site infection	6	4.3
Pneumonia	2	1.4
Cholangitis	2	1.4
Osteomyelitis	2	1.4
Ventriculitis	2	1.4
Not significant	53	38.1
Asymptomatic bacteriuria	23	16.5
Respiratory colonization	11	7.9
Another colonization	19	13.7
**Sample** **source**		
Urine	66	47.5
Peritoneal fluid or other abdominal source	20	14.4
Respiratory simple	17	12.2
Blood	15	10.8
Abscesses	4	2.8
Cerebrospinal fluid	2	1.4
Others	15	10.8
**Antibiotic** **treatment** **for** **significant** **events**		
Combination therapy	60	43.2
Carbapenem + polymyxin	29	20.9
Carbapenem + aminoglycoside + fosfomycin	10	7.2
Carbapenem + fosfomycin	6	4.3
Carbepenem + tigecycline	3	2.1
Carbapenem + aminoglycoside	3	2.1
Carbapenem + tigecycline + ciprofloxacin	3	2.1
Carbapenem + tigecycline + polymyxin	3	2.1
Aminoglycoside + Fosfomycin	2	1.4
Monotherapy	23	16.5
Carbapenem	19	13.7
Fosfomycin	2	1.4
Polymyxin	2	1.4

The main cause of hospitalization was surgical intervention for 35 patients (51.4%) and the most frequent procedures were related with gastrointestinal tract, and liver transplant for 17 (25.0%) and 9 (13.2%) patients, respectively. The average time between admission to the institution and the appearance of the first KPC-*Kp* isolate was of 8 days with a range of − 4 to 748 (negative value indicates a patient that was admitted after a screening for colonization) and an average hospital stay of 27 days and the median hospital stay was 29 days, being higher in patients with more than one event compared to patients with only one isolate (56 vs 25 days, 28 vs 41 patients, respectively; p < 0.01). During hospital stay, 45 (66.2%) patients had admission to the Intensive Care Unit, with an average APACHE of 18 (ranging from 5 to 26) and SOFA of 6 (range 1–18). Finally, 12 (17.6%) patients died during the study period, three (4.4%) attributable to sepsis due to *K.* *pneumoniae* (Table 1).

Although most of the patients (41, 60.3%) had a unique KPC-*Kp* event, 27 patients had recurrent KPC-*Kp* isolates, with 2 (n = 15, 55.5%), 3 (n = 5, 18.5%), 4 (n = 1, 3.7%) and > 5 (n = 6, 22.2%) isolation events. Notably, there were two cases with 13 and 16 KPC-*Kp* isolates, respectively. The average of KPC-*Kp* isolates in each patient was 2.06. In 54 (79.4%) patients, the first or unique KPC-*Kp* isolate caused health care-associated infections/events (HCA); eight patients (11.8%) had hospital acquired (HA) infections/events (referred from other institutions) and six (8.8%) patients had community-acquired infections/events (CA). Most common infection processes associated to these KPC-*Kp* isolates were urinary tract infection and bacteremia for 42 (30.2%) and 17 (12.2%) patients respectively (Table 2). Regarding the antibiotic treatment for the infectious events, 86 (61.9%) were clinically significant of which 60 (43.2%) had treatment with combination therapy and 23 (16.5%) monotherapies. A carbapenem monotherapy treatment was observed in 19 (13.7%) events, and a combination therapy with some carbapenem in 57 (41.0%) of these, 29 (20.9%) were with polymixin (Table 2).

### Intra and inter-patient variation of antibiotic susceptibility profiles

The isolates presented 16 different susceptibility profiles for non-β-lactam antibiotics (Fig. S1). Eighty nine (64.0%, n = 139) isolates were resistant to ciprofloxacin, 52 (37.4%, n = 139) to gentamicin, 19 (13.7%, n = 139) to amikacin, 7 (8.2%, n = 85) to colistin, and 3 (5.1%, n = 59) to tigecycline. In the 27 patients with recurrent cases (two or more events), only 11 (40.7%) kept the same resistant profile during the different events, suggesting the persistence of the same bacterial population over time. On the other hand, remaining 16 (59.3%) patients with recurrent events, showed changes in the resistance profiles from different events, suggestive of differential acquisition of resistance (by SNPs or Horizontal Gene Transfer) or recurrent events due to different clone populations (Fig. S1). None isolate harbored the *bla*_GES_, *bla*_NDM_, *bla*_IMP_, *bla*_OXA-48_ and *bla*_VIM_ genes. However, the *bla*_TEM_ and *bla*_CTX-M_ genes were found in 53 (38.1%) and 39 (28.1%) isolates, respectively; and 29 (20.9%) isolates harbored both genes simultaneously.

### Broad genetic diversity of isolates

**Figure 1:**
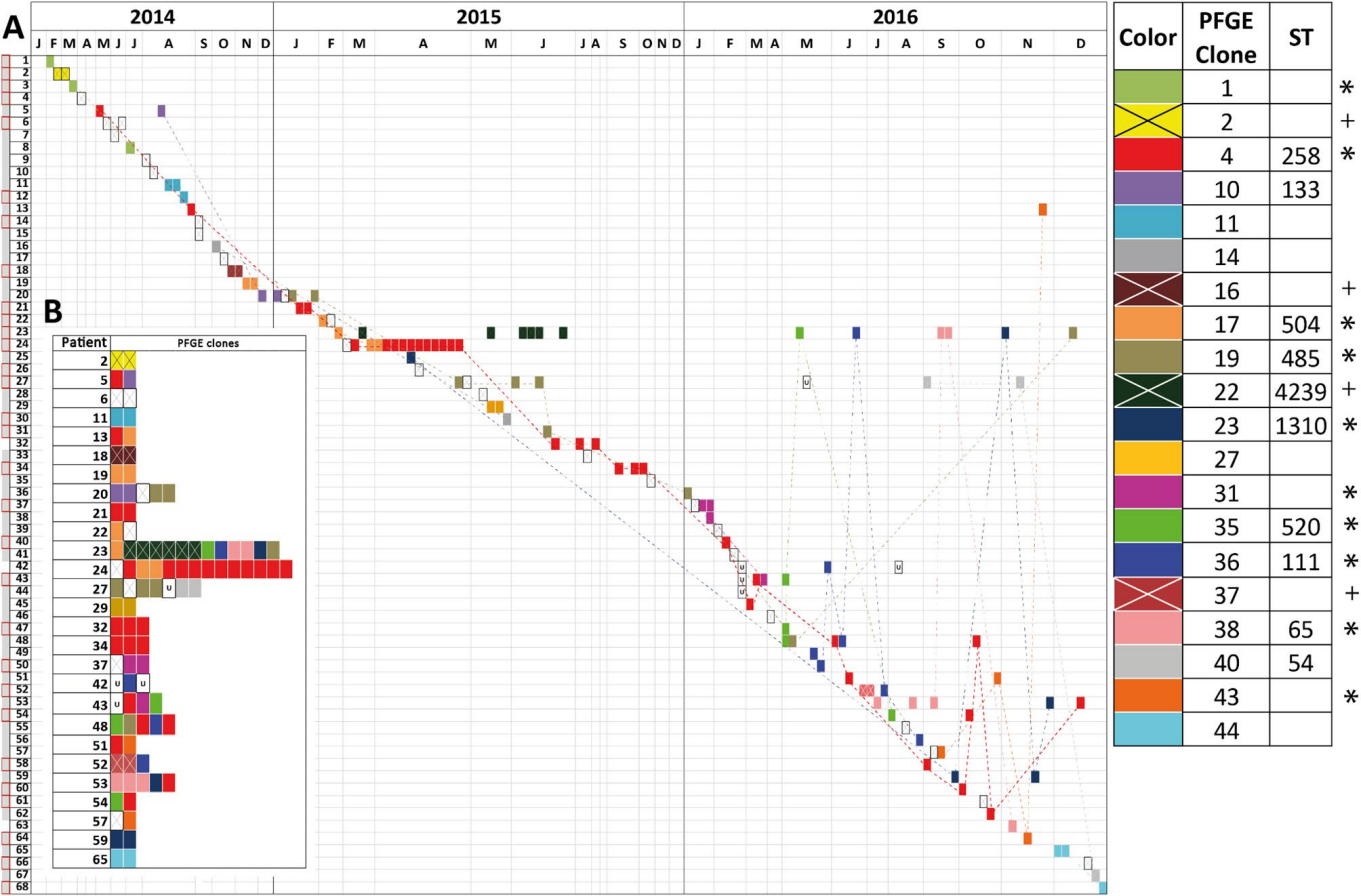
Circulation dynamics of the clones of *Klebsiella* *pneumoniae* carrying *bla*_KPC_ gene in one complex health institution during 3 years. (**A**) On the left side are the patients and above the months of the respective years. The gray boxes next to the number of each patient indicate the prior antibiotic treatment to first KPC-*Kp* event, and were highlighted with a red border, those who had carbapenems among the therapeutic options used. The white rectangles with a X indicate the 25 clones that were identified in a single event, and colored rectangles indicate the 20 clones that were identified in two or more events (see upper right box for more information). The boxes marked with a "U" indicate those isolates in which it was not possible to identify the pulsotype and therefore the associated clone is unknown. The legend specifies the clones that were detected in a single patient (+) or in more than three patients (*), and the dotted lines indicate the order of appearance of the clones isolates from two or more patients. (**B**) It only illustrates in colored boxes KPC-*Kp* isolates of patients who had more than one event, which allows us to observe intra-patient clonal variability.

Regarding the genetic relationship, 45 different clones were identified through PFGE analysis of all 139 isolates, which suggests a polyclonal behavior in the institution (Fig. S2). The most frequent clones were 4, 19 and 36, identified in 32 (23.0%), 9 (6.5%) and 8 (5.7%) isolates respectively. The sequence type (ST) was determined in 12 clones, including the clones with the highest circulation. The STs identified were: ST258 (clone 4), associated with the pandemic clone with the highest circulation, ST54 (clone 40), ST133 (clone 10), ST1310 (clone 23), clones belonging to the CC65 (ST65/clone 38, ST520/clone 35, ST846/clone 21, and ST111/clone 36), and CC485 (ST485/clone 19, ST504/clone 17, ST4239/clone 22, and ST4959/clone 18) (Fig. 1, Fig. S3). These results showed that, although the pandemic clone ST258 (associated to clone 4 and with 23.0%) was the most frequent, the other non-CC258 clones have notoriously increased its frequency, and perhaps with different genetic characteristics harboring *bla*_KPC_ gene, which could impact dynamics of transmission. Interestingly, ST258, ST65 and ST133 were the only clones that had been previously reported with *bla*_KPC_; while for the remaining nine STs (ST54, ST1310, ST520, ST846, ST111, ST485, ST504, ST4239 and ST4959) this is the first world report in which these are described harboring *bla*_KPC,_ showing other *K.* *pneumoniae* clones are acquiring this important gene^13,41–47^.

Analysis of clonal KPC-*Kp* circulation dynamics in the 41 patients with only one isolation event, showed 17 (41.5%) had exclusive clones (Fig. 1, marked with an X) and 23 (56.1%) presented inter-patient disseminated clones (including the clone 4/ST258). In the 27 patients having multiple isolation events, there were 12 clones that exclusively presented intra-patient dissemination (Fig. 1, marked with an X), four of them generated relapses in the same patient (clones 2, 16, 22 and 37). In this group of relapsing patients there were 16 clones with inter-patient dissemination (including the clone 4/ST258), all of them but one (clone 43) were also associated to relapse/reinfection in at least one patient (see Fig. 1B). In conclusion, CC258 related and not related KPC-*Kp* isolates were associated to intra- and inter-patient dissemination. However, the risk of a relapse was about three times higher in CC258 vs non-CC258 patients (odd ratio 3.143, with a 95% confidence interval ranging from 1.033 to 9.560). A comparison of the clinical characteristics of the patients who had non-CC258 vs CC258, showed the last one was associated to older patients (52.0 vs 69.5 y.o., in non-CC258 vs CC258, respectively, p = 0.03) and longer average hospital stay (57 vs 110 days, in non-CG-258 vs CG-258, respectively, p = 0.624) (Table S4).

### Inter- and intra-patient clonal dynamics

Despite CC258 was not predominant among the 139 isolates (only accounted for 23.0% of the isolation events), it was the only one that prevailed over the 3 years of study, revalidating the success of this pandemic clone (Fig. 1). There were other clones with inter-patient dissemination supported by the close temporal proximity in their collection, but most of them in the third year of study and associated to short intra-hospital appearances (Fig. 1, denoted by dashed lines). To try to understand the possible mechanisms involved in the intra-patient clonal variation we further reviewed in the four patients (23, 24, 27 and 48) that had more than five isolates with more than four PFGE clones. Interestingly, some clones in the same patient exhibited different resistance profiles, such as clone 17, 22, 40 and 19 (all non CC258) in patients 24, 23 and 27; and clone 4 (CC258) in patients 24 and 48 (Fig. 1B, Fig. S1). These results suggest resistance evolution in isolates generating multiple events in the same patient, although reinfection by multiple clones closely related cannot be ruled out.

### Reinfection dynamics of patient 24 over her hospital stay

**Figure 2:**
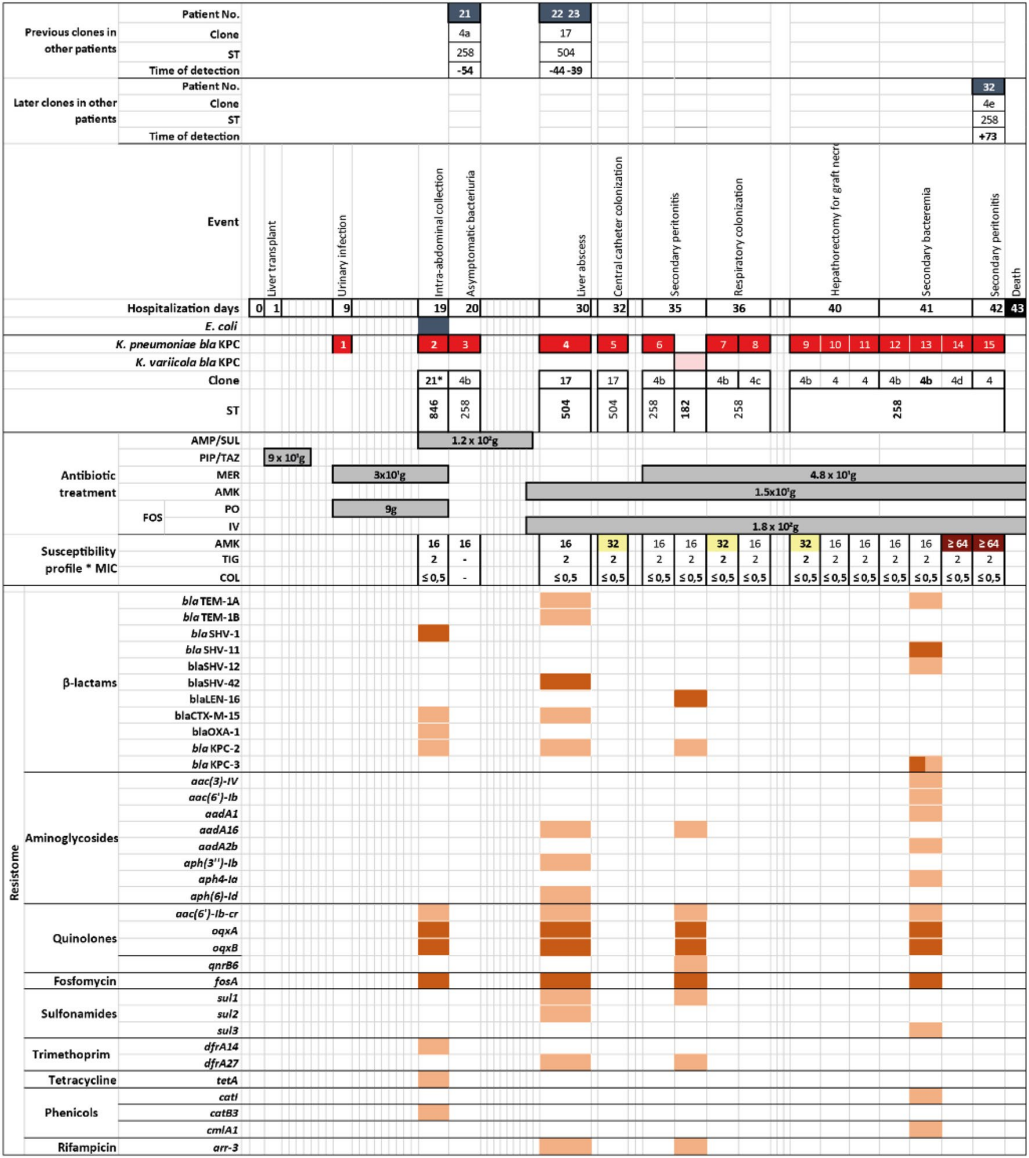
Analysis of the circulation dynamics of KPC-*Kp* clones of patient number 24 during antibiotic treatment in the hospital stay in 2015. The graph illustrates to scale the timeline from the admission of the patient to the institution (day 0) until death (day 43), with a description of the events associated with the KPC-producing *K.* *pneumoniae* (KPC -*Kp)* isolates 33Kpn9, 33Kpn12 and 33Kpn22 which were red colored respectively; and a pink box for KPC-producing *K.* *variicola* (KPC-*Kv*) isolate 33Kva16, with their respective MLST. Regarding the susceptibility profile, only the antibiotics to which the isolates were susceptible were illustrated, within the boxes the value in white, yellow and dark red of the minimum inhibitory concentrations (MIC) corresponding to the range of the points of inhibition are described. CLSI cutoffs: susceptible, intermediate, and resistant, respectively. The type of antibiotic used with the cumulative dose in grams and the days of duration of treatment are described in gray boxes. Regarding the resistome of the sequenced isolates, the genes present were organized considering the type of antibiotic and shaded in light orange if they had plasmid location and in dark if it was chromosomal, chromosomal encoded beta-lactamase *bla*_SHV_ or *bla*_LEN_ were not shown. Finally, the connection with other patients who presented the same clone and the number of previous (−) or later (+) days of difference are shown.

To try to understand the possible mechanisms involved in the patient reinfection dynamics, we used whole genome sequencing to analyze *bla*_KPC_ dissemination in the patient that presented the highest number of isolation events in the study (patient 24), a 54-year-old woman who was treated in 2015 and had a hospital stay of 43 days. She was admitted for liver transplant with a diagnosis of primary biliary cirrhosis and 14 KPC-*Kp* isolates were recovered, which were associated with three different clones: 4 (ST258), 17 (ST504) and 21 (ST846) and curiously a *K.* *variicola* (ST182) isolate, harboring *bla*_KPC,_ was also isolated during KPC-*Kp* events. Although changes in the susceptibility profiles of the isolates were not associated with specific clones; in the initial events, the isolates presented susceptibility to aminoglycosides and as the selective pressure to these antibiotics continued, there was a progressive increase in the minimum inhibitory concentration (MIC) until resistance was reached (Fig. 2). Interestingly, the clone that predominated during the hospital stay was ST258, however, this appeared after carbapenem use, like patient 48 where CC258 related clones appeared also after a carbapenem was administered (Fig. S6). Patient 24 finally died due to a KPC-*Kp* attributable infection. *K.* *pneumoniae* *i*solates 33Kpn22 (ST258), 33Kpn12 (ST504) and 33Kpn9 (ST846), representing clones 4, 17 and 21; and *K.* *variicola* 33Kva16 (ST182) isolate, all found in patient 24 were sequenced and the resistome and *bla*_KPC_ related mobilization platforms were studied.

### Resistome of KPC-*Kp* clones

Genome sequencing confirmed that the three clones were genetically different, as observed in the PFGE and MLST analysis. Assembly of the libraries resolved the complete structures of the chromosomes and plasmids, and the presence of resistance genes. In detail, the 33Kpn9 (ST846) isolate had a chromosome of 5.5 Mbp and harbored two plasmids, p33Kpn9-KPC (IncFII(K)) and p33Kpn9-2 (IncFIB(K)) of 133 and 125 Kbp respectively. Its resistome was formed for ten genes: *fosA* and *oqxAB* in chromosome, and *bla*_CTX-M_, *bla*_OXA-1_, *bla*_KPC-2_, *aac(6* *')-Ib-cr*, *drfA14*, *tetA,* and *catB3* in the plasmid p33Kpn9-KPC. The 33Kpn12 (ST504) isolate had a chromosome of 5.3 Mbp and two plasmids: p33Kpn12-1 (IncN) and p33Kpn12-KPC (ColRNAI) of 117 and 16 Kbp respectively. This isolate presented 14 resistance genes: *fosA* and *oqxAB* on chromosome, *bla*_TEM-1_, *bla*_CTX-M-15_, *aadA16*, *aph(6)-Id*, *aac(6* *')-Ib-cr*, *drfA27*, *sul1*, *sul2* and *arr3* in plasmid p33Kpn12-1; and *bla*_TEM-1_ and *bla*_KPC-2_ in p33Kpn12-KPC. The 33Kpn22 (ST258) isolate had a chromosome of 5,5 Mbp and five plasmids: p33Kpn22-1 (IncF), p33Kpn22-2, p33Kpn22-3 (IncFII(Yp)), p33Kpn22-KPC (IncI2 (Δ)) and p33Kpn22-5 (IncR), with 197, 175, 108, 75, and 47 Kbp. From this isolate 20 resistance genes were identified: *bla*_KPC-3_, *fosA* and *oqxAB* on the chromosome; *catA1* in p33Kpn22-1; *bla*_SHV-12_, *aac(6')-Ib* (two copies) and *aac(6')-Ib-cr* (two copies) in p33Kpn22-3; *bla*_TEM-1A_ and *bla*_KPC-3_ in p33Kpn22-KPC; and *aac(3)-IV*, *aac(6')-Ib*, *aadA1*, *aadA2b*, *aph(4)-Ia*, *cmlA1*, *aac(6')-Ib-cr* and *sul3* in p33Kpn22-5 (table S3). To summarize, *bla*_KPC_ was found in a single plasmid copy in all sequenced isolates, but 33Kpn22 (ST258) had an additional chromosomal copy. Regarding with KPC-*Kv* isolate, 33Kva16 (ST182) had a partial assembled chromosome distributed in seven contigs and two whole assembled plasmids: p33Kva16-1 (IncFIB(K)) and p33Kva16-KPC (IncN) of 122 and 58 Kbp respectively. This isolate presented 10 resistance genes: *oqxAB*, *fosA*, in the chromosome; and *bla*_KPC-2,_ *aadA16*, *aac(6* *')-Ib-cr*, *qnrB6*, *sul1*, *drfA27*, and *arr3* in plasmid p33Kva16-KPC.

Comparison of the resistome profiles of the three KPC-*Kp* and KPC-*Kv* sequenced isolates with the antibiotic therapy in patient 24 showed the isolates had resistance mechanisms to all the therapeutic options used, which surely hampered the success of the treatment. In the case of β-lactams, in addition to the previously identified *bla*_KPC_, genes encoding for TEM, CTX-M and OXA were also found. Of note, *fosA* gene, conferring resistance to phosphomycin, was in the chromosome of all isolates, and this antibiotic was used during most of the hospital stay. In the case of aminoglycosides, it was observed that, during the selective pressure exerted with the administration of amikacin, the clones with the highest number of genes for resistance to this antibiotic were selected (Fig. 2).

### Tn***3***-based transposons are responsible of ***bla***_KPC_ mobilization

**Figure 3:**
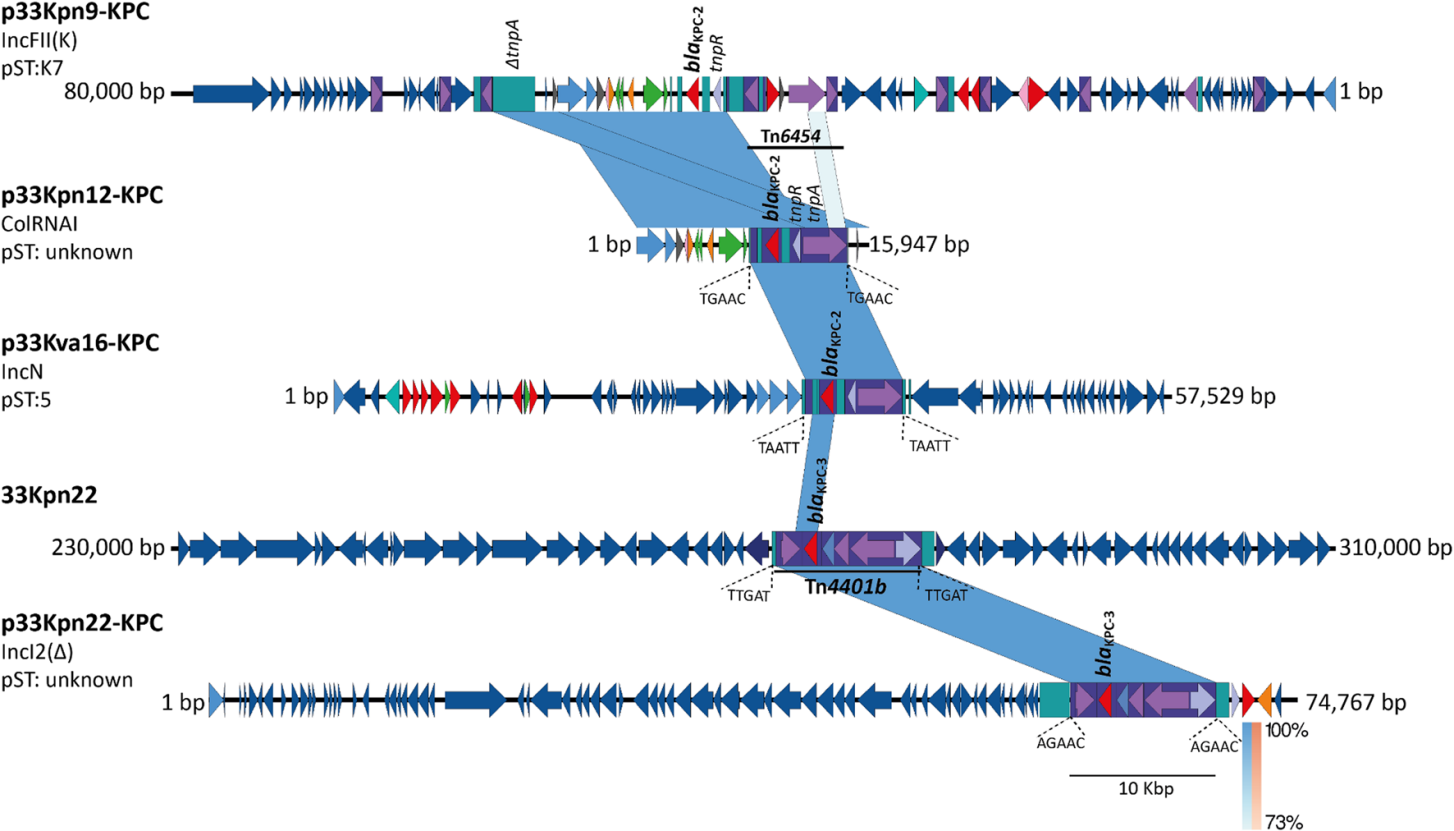
Comparative genomic analysis of *Klebsiella* sequences that harbored *bla*_KPC_ gene isolated from patient 24. p33Kpn9-KPC (CP064297), p33Kpn12-KPC (CP062794), 33Kpn22 (CP069046) and p33Kpn22-KPC (CP069050) for *K.* *pneumoniae*, and p33Kva16-KPC (JAGTWT010000009) for *Klebsiella* *variicola*. The shaded area between the sequences delimits the alignment regions with a percentage identity ≥ 73%. The blue arrows indicate the open reading frames and, adjacent to the gene of interest, the coding regions for hypothetical proteins (gray), transposases (purple), resolvases (purple), resistance genes (red) are highlighted and delimited by a purple rectangle to transposons. The duplication sites (TSDs) generated by the transposition were illustrated and delimited in dotted lines for each mobile genetic element. The graph has a scale line of 10,000 bp.

Comparative analysis of *bla*_KPC_ adjacent regions in the sequences of these three KPC-*Kp* isolates established two Tn*3*-like transposons: Tn*4401b* and Tn*6454* (a new genetic structure identified here and registered in The Transposon Registry) and which were involved in the acquisition of this resistance gene (Fig. 3). The new putative transposon Tn*6454* was identified in the KPC-Kp isolates p33Kpn12-KPC, p33Kpn9-KPC (all non-CC258) and KPC-*Kv* isolate p33Kva16-KPC; and corresponds to a new isoform (e) of the NTE_KPC-_II elements. These elements can be classified in the groups I, II or III, where the presence of *bla*_TEM_ upstream *bla*_KPC_ is a distinctive mark for the members of group II. In the transposon Tn*6454* there is a Δ*bla*_TEM_, hence this new transposon belongs to group II. Genetic structure of this new NTE_KPC_-II is not related to any of the previously reported subgroups (a–d). For this reason, we propose here Tn*6454*-related transposons can be described as a new subgroup NTE_KPC_-IIe.

**Figure 4:**
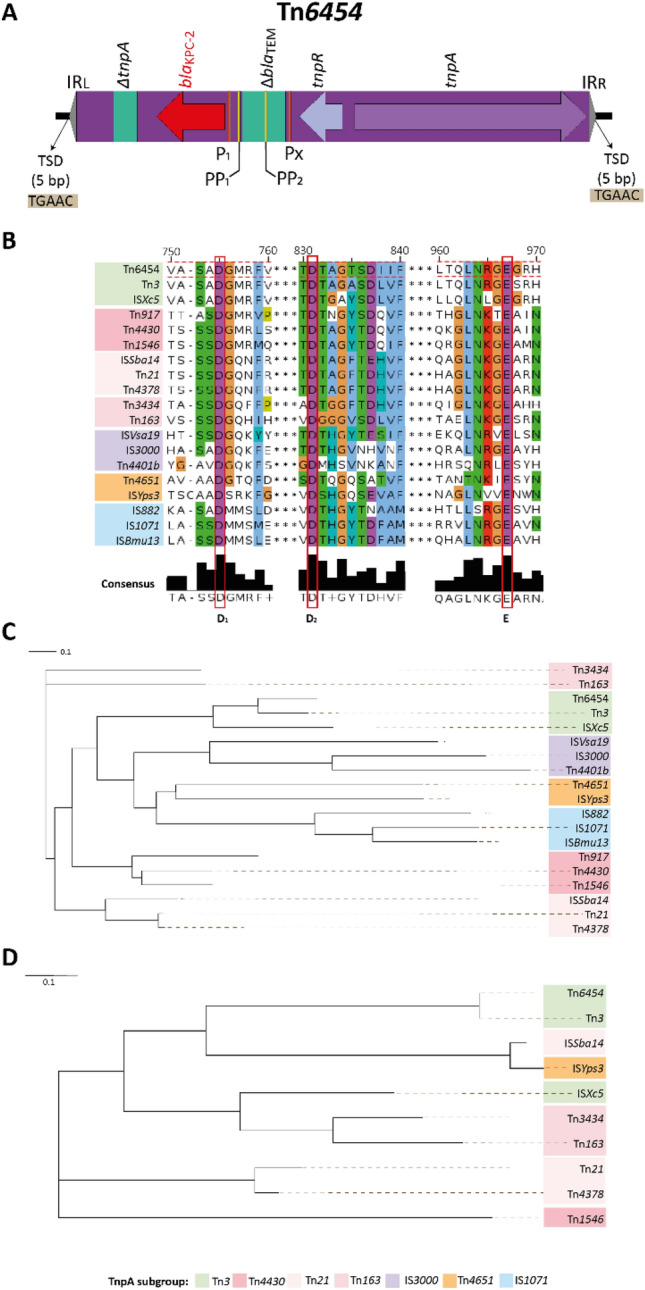
Tn*6454* characterization. (**A**) General structure of Tn*6454*. The arrows indicate the open reading frames, the coding regions for transposases (purple), resolvases (lilac), resistance genes (red) and truncated genes (green) are highlighted, the transposons are delimited in a purple rectangle. The inverted repeats are little gray arrows and the duplication sites (TSDs) generated by the transposition were illustrated inside a gray rectangle. Promoters of *bla*_KPC_ gene are indicated by orange and yellow lines, the orange lines indicate the promoters P1 AND PX and the yellow lines the putative promoters PP1 (− 10 ttttaattt, − 35 ttgatt) and PP2 (− 10 taatagact, − 35 tagctt) with a linear discriminant function (LDF) of 7.99 and 0.78, respectively. (**B**) Multiple sequence alignment of Tn*3*-family transposase proteins, the numbers correspond to alignment positions in aa and the dotted lines showed the DDE motif. Insertion sequences representative of diversity of IS*Kra4* family are indicated on the left. (C) Phylogenetic NJ tree of the Tn*3*-family transposase proteins and (D) S-recombinase family proposed by Nicolas et al.^37^. The different clusters and subgroups identified within the family are boxed with different colors as indicated. The length of the branches is proportional to the average number of substitutions per residue.

The Tn*6454* is flanked by the IRs GGGGTTTGAAGGCCAATGGAACGAAAACGTACGTTAAGA/GGGGTTTGAGGGCCAATGGAACGAAAACGTACG, is composed of *tnpA* and *tnpR* genes, encoding a transposase and a resolvase, needed for the mobilization of the genetic element; and harboring as passenger genes *bla*_KPC-2_, and remains of a ΔIS*Kpn6* and Δ*bla*_TEM_ (Fig. 4A). The insertion of these remains produced an alteration in the promoter sequences of the *bla*_KPC-2_ gene. The bioinformatic analysis detected two new putative promoters (PP1 and PP2), located at − 10 and − 35 regions, additional to previously reported P1 and PX promoters (Fig. 4A). The possible gene expression changes should be assessed.

The *tnpA* of Tn*6454* encodes for a protein of 1,002 amino acids (aa), with 39.6 and 77.4% sequence similarity, and 22.0 and 64.2% sequence identity to the TnpA of Tn*4401b* and Tn*3,* respectively. Furthermore, TnpA of Tn*6454* has the conserved motif of three acidic residues (two aspartic acids and a glutamic, DDE), present in all members of the DDE transposase family, including the Tn*3*-family (Fig. 4B). Phylogenetic analysis with 18 full-length sequences of transposases belonging to Tn*3*-family revealed that TnpA of Tn*6454* grouped to the Tn*3* subgroup while the TnpA of the Tn4*401b* to the IS*3000* subgroup (Fig. 4C). With respect to the cointegrate resolution module, in the Tn*6454* the recombinase belongs to the serine recombinase (S-recombinase) family and is identical to the Tn*3* resolvase (100% sequence identity and coverage) (Fig. 4D). In summary, we are reporting here a new Tn*3*-family platform responsible of *bla*_KPC-2_ mobilization in non-CC258 KPC-*Kp* isolates.

Regarding the Tn*4401b* in patient 24 (isolate 33Kpn22, CC258), this harbored a *bla*_KPC-3_ gene variant and was identified in both the chromosome and the p33Kpn22-KPC plasmid (Fig. 3, S7), suggesting a mobilization of this transposon, widely described in the literature, that allows the stabilization of the resistance mechanism in the bacteria progeny. To determine whether these transposons were the platforms that promoted the *bla*_KPC_ establishment in the plasmids and chromosome of the isolates, an analysis of the target site duplication (TSD) was carried out and five identical nucleotides flanking the transposons were observed in all Tn*3-*based structures, Tn*6454* and Tn*4401b* (Fig. 3); reinforcing the capability of this platforms to mobilize *bla*_KPC_.

### Comparative genomics of the plasmids harboring the Tn*6454* in *Enterobacteriaceae* isolates

**Figure 5:**
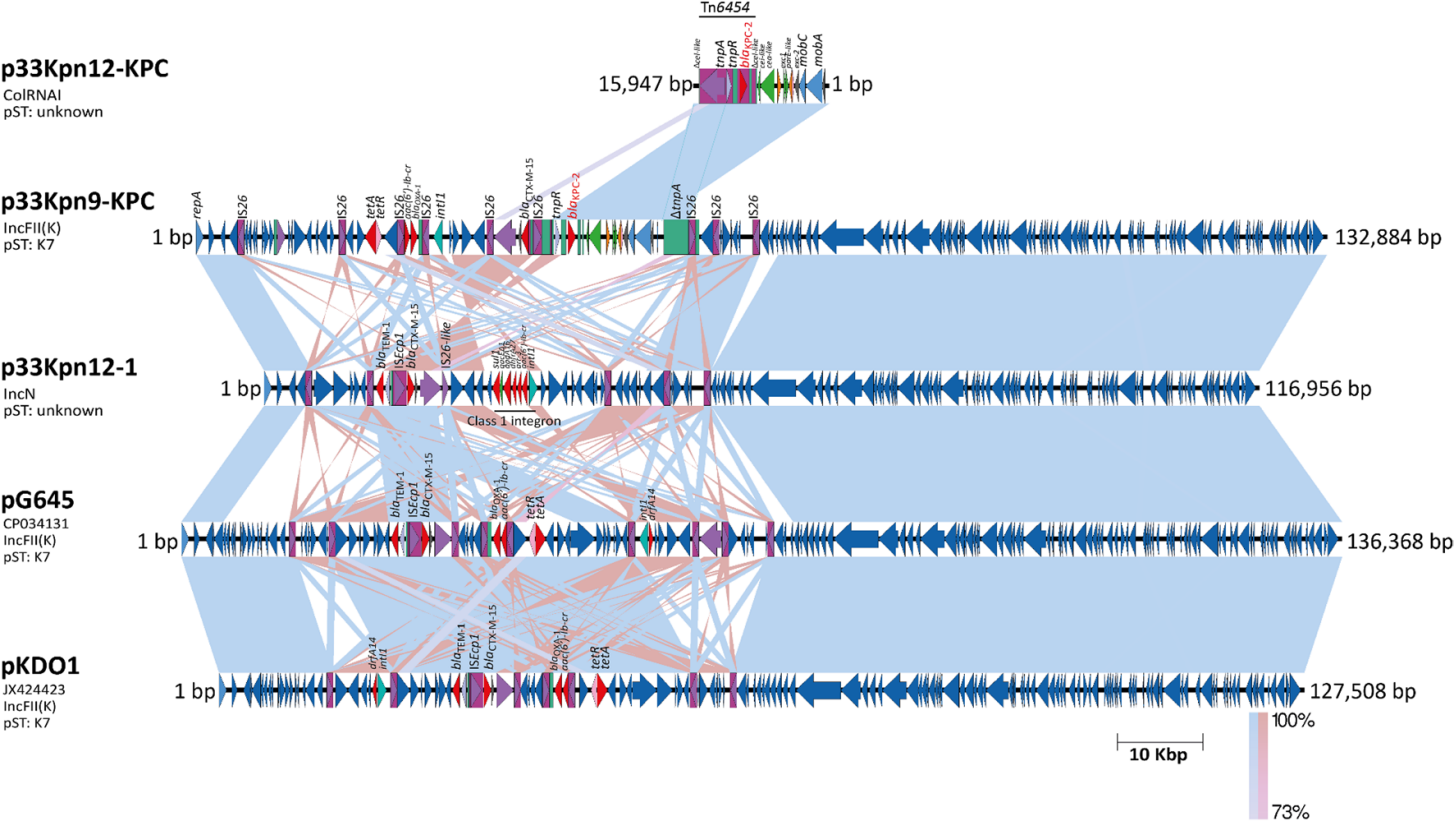
Comparison of the plasmids determined in the *K.* *pneumoniae* 33Kpn9 and 33Kpn12 isolates with the IncFII (K) plasmids previously reported. The sequences p33Kpn9-KPC (CP064297), p33Kpn12-1 (CP062793) and p33Kpn12-KPC (CP062794) were compared using a BLAST Pairwise Alignment with Nucleotide database, pG645 (CP034131) of *K.* *quasipneumoniae* and pKDO1 (JX424423) of *K.* *pneumoniae* were the plasmids with more identity percent. The shaded area between the sequences delimits the alignment regions with a percentage of identity ≥ 73%. The blue arrows indicate the open reading frames and, adjacent to the gene of interest, the coding regions for hypothetical proteins (gray), transposases (purple), resolvases (purple), resistance genes (red) are highlighted and delimited by a purple rectangle to transposons. The duplication sites (TSDs) generated by the transposition were illustrated and delimited in dotted lines for each mobile genetic element. The graph has a scale line of 10,000 bp.

Although there was no relation between plasmids harboring *bla*_KPC_ in the three KPC-*Kp* isolates, the almost complete sequence (99.7%) of the small plasmid p33Kpn12-KPC (16Kbp) was found cointegrated to a bigger plasmid in the isolate 33Kpn9 (Figs. 3, 5). This cointegrated plasmid (p33Kpn9-KPC) belongs to the IncFII(K) incompatibility group, with a conserved backbone covering > 80% of the plasmid and reported previously in other plasmids of this group (such as pG4584 and pKDO1, Fig. 5).

Interestingly, a similar *bla*_KPC_-negative plasmid was also found in the isolate 33Kpn12 (plasmid p33Kpn12-1, Fig. 5); however, this plasmid did not have the hallmark of the IncFII(K) group. The presence of the plasmid p33Kpn12-1 (IncFII(K)-like) and the small p33Kpn12-KPC in the same isolate, further support the possibility of cointegration between these structures.

**Figure 6:**
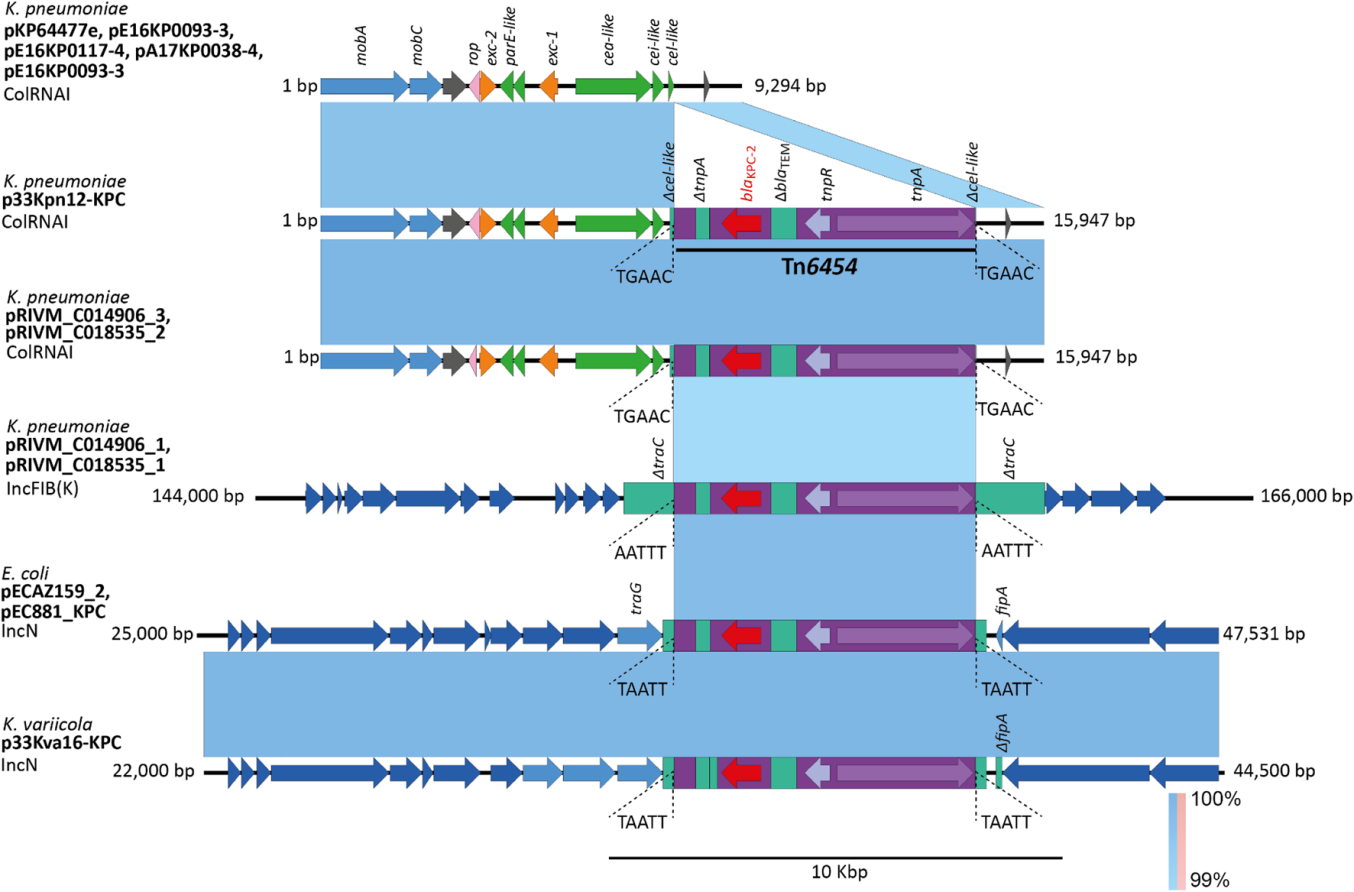
Comparative genomics of the plasmid p33Kpn12-KPC (CP062794) (ColRNAI) and p33Kva16-KPC (JAGTWT010000009) (IncN) harboring the new transposon Tn*6454*, a genetic element previously found in *K.* *pneumoniae* ColRNAI and IncFIB plasmids, and *E.* *coli* IncN plasmids. The shaded area between the sequences delimits the alignment regions with a percentage identity ≥ 99%. The blue arrows indicate the open reading frames and adjacent to the gene of interest, the coding regions for transposases (purple), resolvases (lilac), resistance genes (red) are highlighted, the transposons are delimited in a purple rectangle. The duplication sites (TSDs) generated by the transposition were illustrated and delimited in dotted lines for each mobile genetic element. The graph has a scale line of 10,000 bp.

The small plasmid p33Kpn12-KPC seem to be important for the *bla*_KPC-2_ acquisition in *K.* *pneumoniae* isolates. This plasmid was classified as ColRNAI and a comparison of the complete sequence of this plasmid with the NCBI Nucleotide database yielded more than thirty entries (revised 02-26-2021); most of them corresponded to the naïve plasmid, without resistance determinants, reported in *K.* *pneumoniae* and other *Enterobacteriaceae* in distant regions, such as Brazil, Korea, and Czech Republic. Remarkably, there were two identical plasmids (pRIVM_C014906_3 and pRIVM_C018535_2), harboring the Tn*6454* reported in two KPN-*Kp* isolates from The Netherlands (Fig. 6)^48^. These Dutch isolates also harbored another plasmid IncFIB(K) (pRIVM_C014906_1 and pRIVM_C018535_1) with a Tn*6454* copy.

Tn*6454* sequence of *K.* *pneumoniae* plasmids and *K.* *variicola* IncN plasmid was found in two additional IncN plasmids from two Colombian *E.* *coli* isolates: pECAZ159_2 and pEC881_KPC (Fig. 6). Similarly, TSD analysis of these plasmids showed a 5 bp duplication supporting acquisition through a transposition event. These results highlight the Tn*6454* functionality as a new local transposon for *bla*_KPC_ acquisition among *Enterobacteriaceae.* Interestingly, none of the KPC-*Kp* isolates harboring Tn*6454*, reported here or elsewhere belonged to the CC258.

## Discussion

In this study, the population with KPC-*Kp* events presented risk factors previously described such as a high rate of comorbidity, pharmacological immunosuppression (corticosteroids, organ transplantation) and frequent use of broad-spectrum antibiotics^49,50^. More than half of the patients had several events due to KPC-*Kp* during their hospitalization and with a wide range in their appearance, requiring frequent use of broad-spectrum antibiotics and longer hospital stays, which could determine changes in the dynamics of the circulation of clones in the institution and even more interesting in the same patient.

In the hospital institution, we found a polyclonal behavior of KPC-*Kp* isolates, 23.0% belonging to CC258, and 77.0% non-CC258, showing that *bla*_KPC_ gene disseminates into different *K.* *pneumoniae* lineages not only through a clonal spread. However, time course surveillance showed CC258 clone was the only present during the 3 years of study but it was not the predominant clone in the institution contrary to the worldwide molecular epidemiology of KPC-*Kp* that has been associated with the widespread distribution of ST258 for many years, a predominant clone of hospital-associated outbreaks^51^. In our set of isolates classified as CC258/clone 4, we found several related PFGE pulsotypes, indicating some genomic changes in this subpopulation, which concur with previous findings, where a hospital-specific genomic adaptation of ST258 isolates contributed to the persistence of this clone for 8 years similar to our results, where persistence was observed for at least 3 years^52^.

On the other hand, our data highlighted the continuous arising of not-related non-CC258 KPC-*Kp* isolates in this population. This finding has been observed previously in another Colombian study, in which 62% of the isolates were non-CC258, with thirty-four different genetic backgrounds, none of them related to the STs reported here^19^. This polyclonal non-CC258 distribution has been also reported in pre-endemic countries such as France and Portugal, where there is a higher prevalence of non-CC258 isolates, mobilizing *bla*_KPC_ through Tn*4401* (isoforms *a*, *b,* and *d*)^53,54^. This suggests highly diverse *bla*_KPC_-positive *K.* *pneumoniae* isolates are constantly emerging, possibly due to the mobilization of multiple genetic platforms, as it has been shown for KPC-*Kp* isolates from Virginia (USA), with different genetic lineages emerging by local horizontal transfer of *bla*_KPC_-positive plasmids^55^. Arising of new KPC-*Kp* lineages by mean of HGT is also supported by our detection of *bla*_KPC_ in previously reported KPC-negative STs such as ST54, ST111, ST485 and ST846.

The clinical importance of ST differentiation has not yet been defined, some authors suggest that the average age is slightly higher in the group of patients with *K.* *pneumoniae* CC258, which is more frequent resistance to other groups of antibiotics such as aminoglycosides and quinolones and the coexistence of *bla*_TEM-1_ and *bla*_CTX-M-15_^19,56^. In our series, when adjusting for clone, patients with at least one event due to CC258 had a higher mean age when compared with those who presented other pulsotypes (69.5 vs 52 years), which suggests differential clinical behaviors and probably affects a group of patients with greater vulnerability. Statistically significant differences were also found in the frequency of resistance to ciprofloxacin, amikacin, and gentamicin, and the coexistence of *bla*_TEM_ and *bla*_CTX-M_ was also more frequent in those events with CC258; these data would support the theory of a genetic advantage of CC258 mediated by the transposon and by multi-resistance genotypes^57^.

Surprisingly, clonal diversity was observed both in the hospital institution and within-patient, being the last one a phenomenon clinically imperceptible and not deeply studied. Probably, because it has been observed that an individual who has received multiple courses of antibiotics, frequent hospitalizations and with risk factors for these microorganisms can remain colonized for up to 1 year^58^. Here we report multiple events in a patient caused by the same clone (CC258) or by different KPC-*Kp* unrelated clones during hospital stay at this institution. Although there have been some previous reports of within-patient variability, most of them have reported evolution of the same clonal population to gain resistance or to overcome host challenges. Jousset et al. reported evolution of unique KPC-*Kp* clones in the same patient due to ST258 or ST512 clonal populations^16,59^. We also found within-patient variation of CC258/clone 4 population, such as the case of patients 24, 32 and 48, having multiple closely related pulsotypes, which suggest adaptability and evolution. Noteworthy, here we also report a wider within-patient variability, not only because of clonal evolution but due to a complex non-CC258 diverse population. This was proven at different levels of resolution. Different resistance profiles and different macro-restriction patterns by PFGE were observed as well as unrelated genomes.

Further analysis was done of the resistome and mobile genetic elements involved in the *bla*_KPC_ acquisition of *K.* *pneumoniae* representative isolates (ST846, ST504, and ST258 clones), recovered from the patient with the highest number of KPC-*Kp* isolates. Curiously, the *bla*_KPC_ gene variant was different, *bla*_KPC-2_ and *bla*_KPC-3_ was associated with non-CC258 and CC258, respectively. Additionally, both the number of acquired resistance genes and plasmids in non-CC258 isolates was lower than those identified in the ST258 isolate. This is an agreement with what has been reported in multiple studies, where the ST258 has been associated with larger number of resistance genes to different antibiotics and with the predominant presence of the *bla*_KPC-3_ gene, in Colombia, Sweden, and Italy, among others^52,60–62^. Interestingly, CC258 clones in our study appeared usually after a carbapenem treatment, suggesting broad spectrum antibiotic selection favors those bacteria with a bigger resistome and with KPC-3 that has shown higher hydrolysis rates for third generation cephalosporins such as ceftazidime (i.e. CC258 clones)^63^.

Co-circulation of *K.* *variicola* and *K.* *pneumoniae* is an odd event, however, is probably unnoticed due to some species of *Klebsiella* complex are commonly misidentified as *K.* *pneumoniae* by biochemical techniques, due their closeness^64^. Interestingly, Garza-Ramos et al., using multiplex-PCR reported co-circulation of *K.* *variicola* and *K.* *pneumoniae* ST258, in a Mexican patient. Although the *K.* *variicola* isolates were susceptible, in our study both species carried *bla*_KPC-2_, highlighting the importance of *Klebsiella* complex in the dissemination of resistance mediated by KPC^65^. *K.* *variicola* harboring *bla*_KPC_ has been reported in countries with high frequency of KPC-producing isolates, such as Colombia, China, United States and Portugal, the last one associated with Tn*4401b*^66–70^.

Regarding *bla*_KPC_ mobilization platforms, we found that *bla*_KPC-2_ was harbored in a new Tn*3*-family NTE_KPC_-IIe transposon, Tn*6454*, found in the plasmids p33Kpn12-KPC (ColRNAI) and p33Kva16-KPC (IncN). Although there are remains of Tn*4401b* in the new Tn*6454*, the promoter region for *bla*_KPC_ expression was affected by the remodeling processes in this new structure. The Tn*6454* only has one of the three promoters (P1) originally reported for the Tn*4401b*^6^. However, analysis of *bla*_KPC-2_-upstream region in this transposon with BPROM revealed three additional promoters, the PX, previously reported in other NTE_KPC_ element^71^, and two putative promoters (PP1 and PP2, Fig. 4). In vitro studies of P1 + PX activity has shown increased expression for *bla*_KPC_ in Tn*4401b* (not in *a*)^71^, yet activity of PP1 and PP2 needs further validation.

Unexpected, this new NTE_KPC_-IIe transposon was found in several plasmid backbones from the incompatibility groups IncN, IncF (I or II) or ColRNAI, in Colombia and other distant regions^48,72^. Showing this platform is currently circulating among non-CC258 KPC-*Kp* and other *Enterobacteriaceae* and can greatly contribute to the dissemination of *bla*_KPC-2_*.* The smallest plasmid backbone harboring Tn*6454* ever reported was the ColRNAI. This plasmid has been found in China, co-existing with *bla*_KPC-2_-positive IncR or IncFII/R plasmids and more recently in Tn*6454*-positive Dutch *K.* *pneumoniae* isolates, with an additional copy of the Tn*6454* integrated to IncFIB(K) plasmids, which endorse actual mobilization ability of this transferable element^48,73–75^. Our bioinformatic analyses further supported the transposition aptness of Tn*6454*, validated by the presence of all molecular requirements and TSD traces in the different identified replicons. Transposition ability of the new NTE_KPC_-IIe summed to the high variability found in the non-CC258 KPC-*Kp* isolates, pose the threat of emergence of new genetic KPC-positive backgrounds. Lack of settlement and low inter-patient dissemination of non-CC258 KPC-*Kp* clones observed here suggest this resistant determinant has not reach a more harmful genetic background, however, highly disseminative new transposable elements increase the risk of emergence of more problematic carbapenem-resistant pathogens.

In Colombia, before KPC-*Kp* introduction in 2005, resistance to β-lactams in *K.* *pneumoniae* was mainly associated to multiple Extended-spectrum β-lactamases (ESBLs)^18^. Later, KPC-*Kp* dissemination has been extensively attributed to CC258 related clones^60,76^. Yet, the first report -in Colombia and South America- of KPC-*Kp*, corresponded to two non-CC258 isolates (ST337 and ST338)^77^. Indeed, retrospective studies have shown that, despite CC258 prevalence, a huge proportion of *bla*_KPC_ -positive isolates belonged to non-CC258 lineages^18,19,69^, like our findings with around 70% of non-CC258 KPC-*Kp* isolates. The acquisition of *bla*_KPC_ in the species was initially related to plasmid mobilized Tn*4401* variants, as found in the ST258 isolate from this study, which surely favored the dispersion of this resistance mechanism not only in this species, but in other enterobacteria of clinical interest. However, since 2011 some *bla*_KPC_ positive isolates, negative for any Tn*4401* variant have been observed, which suggest an early NTE_KPC_ dissemination^6,18,78,79^. Regarding the new NTE_KPC_ reported here, comparative analysis did not render *K.* *pneumoniae* sequences harboring Tn*6454* in Colombia, nonetheless, we did find this element in other enterobacteria in Colombian and The Netherlands^48^, suggesting this platform is relevant to the dispersion of this resistance mechanism in multiple plasmids, in other geographically distant countries. Our NGS-based study, allowed identification of this new platform in a non-*K.* *pneumoniae* isolate, highlighting the relevance of molecular surveillance.

## Conclusion

Undoubtedly, these results expand the knowledge of clonal dynamics not only in a Colombian institution but also within a patient, showing the emergence of multiple resistance profiles and lineages among KPC-*Kp*, with *bla*_KPC_ mobilized through new Tn*3* family transposons. Remarkable, KPC-*Kp* intra-patient genetic variation, driven by unrelated clones instead of adaptation or evolution, highlights the importance of understanding each event in a patient as unique and not as prolonged colonization, which will favor a more successful clinical treatment, and the need to implement successful strategies to screen and detect those patients at admission to prevent further dissemination and continued mixing and evolution of resistant clones. In addition, we report here two behaviors for genetic populations of KPC-*Kp*. Initially a classic CC258 dissemination, driven by highly resistant and persistent closely relate clones, and on the other hand the constant arising of highly diverse non-CC258, harboring less complex resistance profiles… for now.

## Supplementary Information

Supplementary Information.
