# Supplementary material for: Within patient genetic diversity of blaKPC harboring Klebsiellapneumoniae in a Colombian hospital and identification of a new NTEKPC platform
Source: Sci Rep. 2021 Nov 1;11:21409. doi: 10.1038/s41598-021-00887-2 (PMC8560879; doi:10.1038/s41598-021-00887-2)
Supplement: Supplementary file 1 — Supplementary Information. [file 41598_2021_887_MOESM1_ESM.pdf]

Supplementary material.

Within patient genetic diversity of bla_{KPC} harboring *Klebsiella pneumoniae* in a Colombian hospital and identification of a new NTE_{KPC} platform

Table S1. Primers

Name	Sequence (5'-3')	Target gene	NCBI access number: Region	Reference
GN282	CAGCAGACGAACTTCCTG	*khe*	NZ_CP064352.1:c3786054-3785566	This study
GN283	CGAGGTTTACGTCTCAAC			
GN284	TTCTCTGCCGTTTCCAA	*uidA*	CP062774.1:c2776873-2775060	This study
GN285	GCTGTCGGCTTTAACCTCT			
GN286	CGAGTGGCGGCATACATC	*ehe*	CP000653.1:296048-296305	This study
GN287	CGCTGGCTCGCAAAATGG			
GN315	GTTGCTGACTCAAGGTCAT	*wosA*	CP068152.1:3414289-3415251	This study
GN316	AATCTAATGGGGGTAGCTC			
GN317	GTACTTCGGCTAAGCACAG	*pehS*	CP056539.1:4185012-4186988	This study
GN318	GGTTACCTCATCGAACTGAC			
GN309	TCGCCGCATACACTATTCTCAGAATGA	bla_{TEM}	AL513383	[21]
GN310	ACGCTCACCGGCTCCAGATTTAT			
GN311	ATGTGCAGYACCAGTAARGTKATGGC	bla_{CTX-M}	X92506	[21]
GN312	TGGGTRAARTARGTSACCAGAAYCAGCGG			
GN436	TCGCTAAACTCGAACAGG	bla_{KPC}	AY034847	[22]
GN437	TTACTGCCCGTTGACGCCCAATCC			
GN438	TTGGCCTTGCTGTCCTTG	bla_{NDM}	FN396876	[22]
GN439	ACACCAGTGACAATATCACCG			
GN440	CTATTACTGGCAGGGATCG	bla_{GES}	AF156486	[22]
GN441	CCTCTCAATGGTGTGGGT			
GN442	TGTTTTTGGTGGCATCGAT	bla_{OXA-48}	AY236073	[22]
GN443	GTAAMRATGCTTGGTTCGC			
GN444	GAGTGGCTTAATTCTCRATC	bla_{IMP}	AJ223604	[22]
GN445	AACTAYCCAATAYRTAAC			
GN446	GTTTGGTCGCATATCGCAAC	bla_{VIM}	AF317511	[22]
GN447	AATGCGCAGCACCAGGATAG			

Table S2. Mobile elements of Tn3-family

<table>
<tr><th>Mobile element</th><th>Synonym(s)</th><th>Transposon</th><th>Insertion sequence</th><th>Access number</th><th>TnpA subgroup</th><th>S-recombinase family</th><th>Y-recombinase family</th></tr>
<tr><td>Tn3</td><td>TnSwi1, ISSwi1</td><td style="background:gray"></td><td></td><td>HM769901</td><td>Tn3</td><td style="background:gray"></td><td></td></tr>
<tr><td>ISXc5</td><td>ISXc4, TnXc4</td><td></td><td style="background:gray"></td><td>Z73593</td><td>Tn3</td><td style="background:gray"></td><td></td></tr>
<tr><td>Tn6454</td><td></td><td></td><td style="background:gray"></td><td>CP062794</td><td>Tn3</td><td style="background:gray"></td><td></td></tr>
<tr><td>Tn917</td><td></td><td style="background:gray"></td><td></td><td>M11180</td><td>Tn4430</td><td></td><td style="background:gray"></td></tr>
<tr><td>Tn4430</td><td></td><td style="background:gray"></td><td></td><td>X07651</td><td>Tn4430</td><td></td><td style="background:gray"></td></tr>
<tr><td>Tn1546</td><td></td><td style="background:gray"></td><td></td><td>M97297</td><td>Tn4430</td><td style="background:gray"></td><td></td></tr>
<tr><td>ISSba14</td><td>TnSba14</td><td style="background:gray"></td><td></td><td>NC_009052</td><td>Tn21</td><td style="background:gray"></td><td></td></tr>
<tr><td>Tn21</td><td></td><td style="background:gray"></td><td></td><td>AF071413</td><td>Tn21</td><td style="background:gray"></td><td></td></tr>
<tr><td>Tn4378</td><td></td><td style="background:gray"></td><td></td><td>X90708</td><td>Tn21</td><td style="background:gray"></td><td></td></tr>
<tr><td>Tn3434</td><td></td><td style="background:gray"></td><td></td><td>AY232820</td><td>Tn163</td><td style="background:gray"></td><td></td></tr>
<tr><td>Tn163</td><td></td><td style="background:gray"></td><td></td><td>L14931</td><td>Tn163</td><td style="background:gray"></td><td></td></tr>
<tr><td>ISVsa19</td><td></td><td></td><td style="background:gray"></td><td>NC_011314</td><td>IS3000</td><td></td><td></td></tr>
<tr><td>IS3000</td><td></td><td></td><td style="background:gray"></td><td>AF174129</td><td>IS3000</td><td></td><td></td></tr>
<tr><td>Tn4401b</td><td></td><td style="background:gray"></td><td></td><td>KT378598</td><td>IS3000</td><td></td><td style="background:gray"></td></tr>
<tr><td>Tn4651</td><td></td><td style="background:gray"></td><td></td><td>NC_003350</td><td>Tn4651</td><td></td><td></td></tr>
<tr><td>ISYps3</td><td>TnYps3</td><td></td><td style="background:gray"></td><td>NC_011759</td><td>Tn4651</td><td style="background:gray"></td><td></td></tr>
<tr><td>IS882</td><td></td><td></td><td style="background:gray"></td><td>NC_005241</td><td>IS1071</td><td></td><td></td></tr>
<tr><td>IS1071</td><td></td><td></td><td style="background:gray"></td><td>M65135</td><td>IS1071</td><td></td><td></td></tr>
<tr><td>ISBmu13</td><td>ISBusp1</td><td></td><td style="background:gray"></td><td>NC_007509</td><td>IS1071</td><td></td><td></td></tr>
</table>

*Gray boxes indicate the classification of each mobile element.

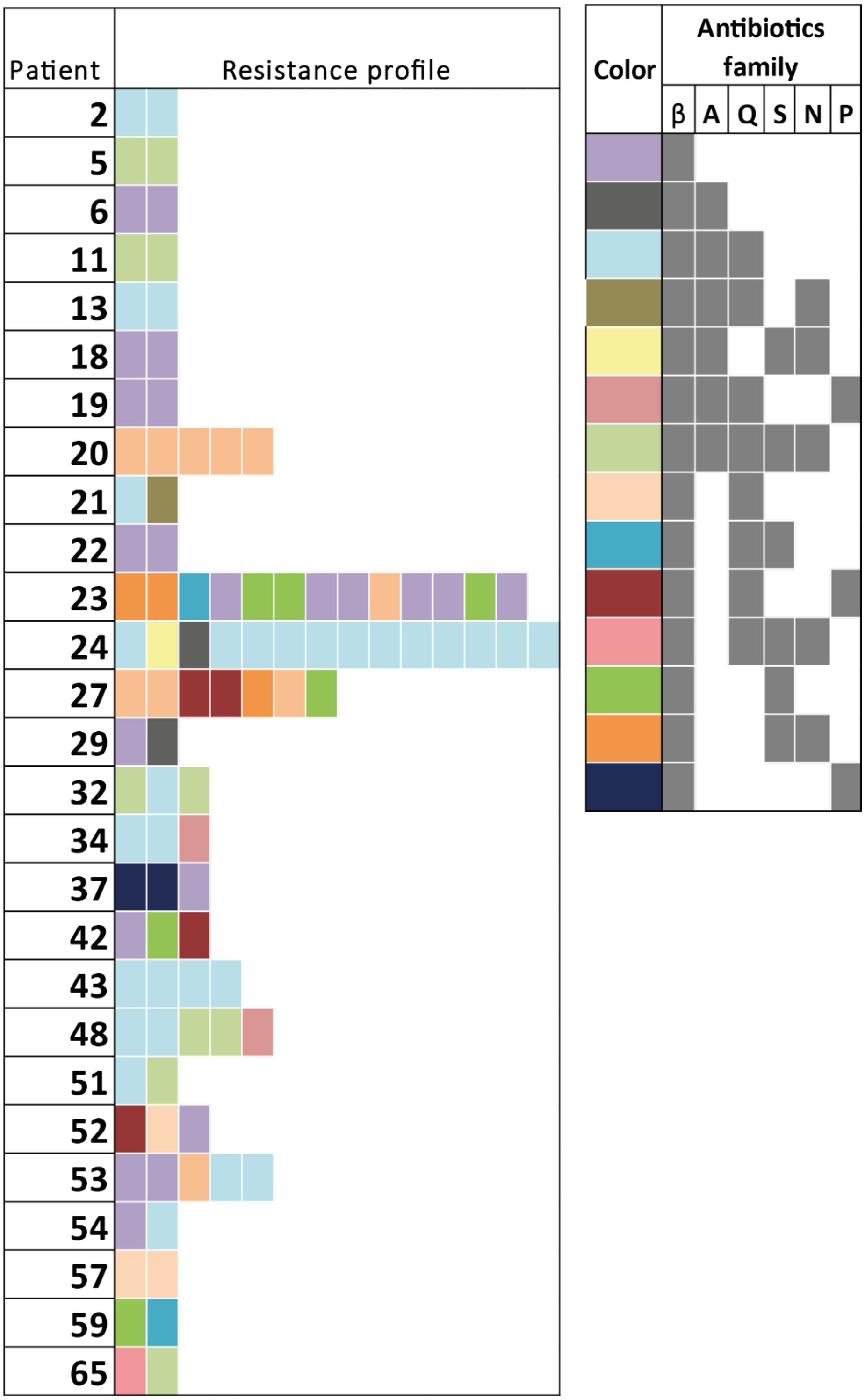

Figure S1. Antibiotic-resistant profiles of patients with multiples (two or more) *Klebsiella pneumoniae* isolates harboring *bla*KPC gene during 2014 to 2016. On the left side is the patient number, colors indicate resistant profiles based in families of antibiotics according to VITEK® 2 Advanced Expert System ™ results, as indicated in the rigth.

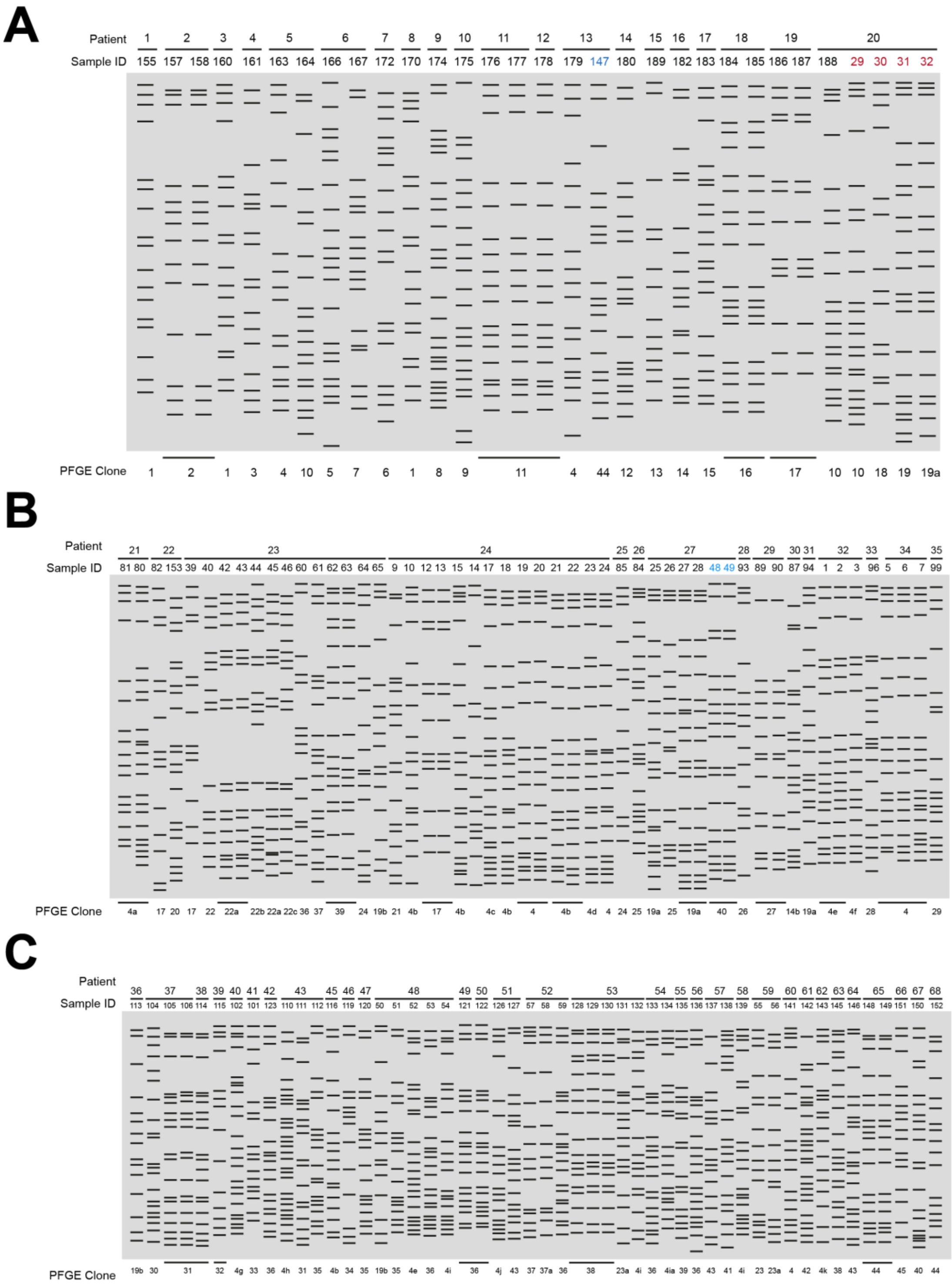

Figure S2. PFGE Clonal distribution by patient of the KPC-*Kp* isolates recovered in (A) 2014, (B) 2015 and (C) 2016. Each Pulsed-field gel electrophoresis (PFGE) pulsotype was illustrated and ordered by patient and date. The first line represents the patient number, second line the sample ID, third line the pulsotype and the last one the PFGE clone. The blue numbers in sample ID represent isolates from 2016 in patients with isolates from previous years.

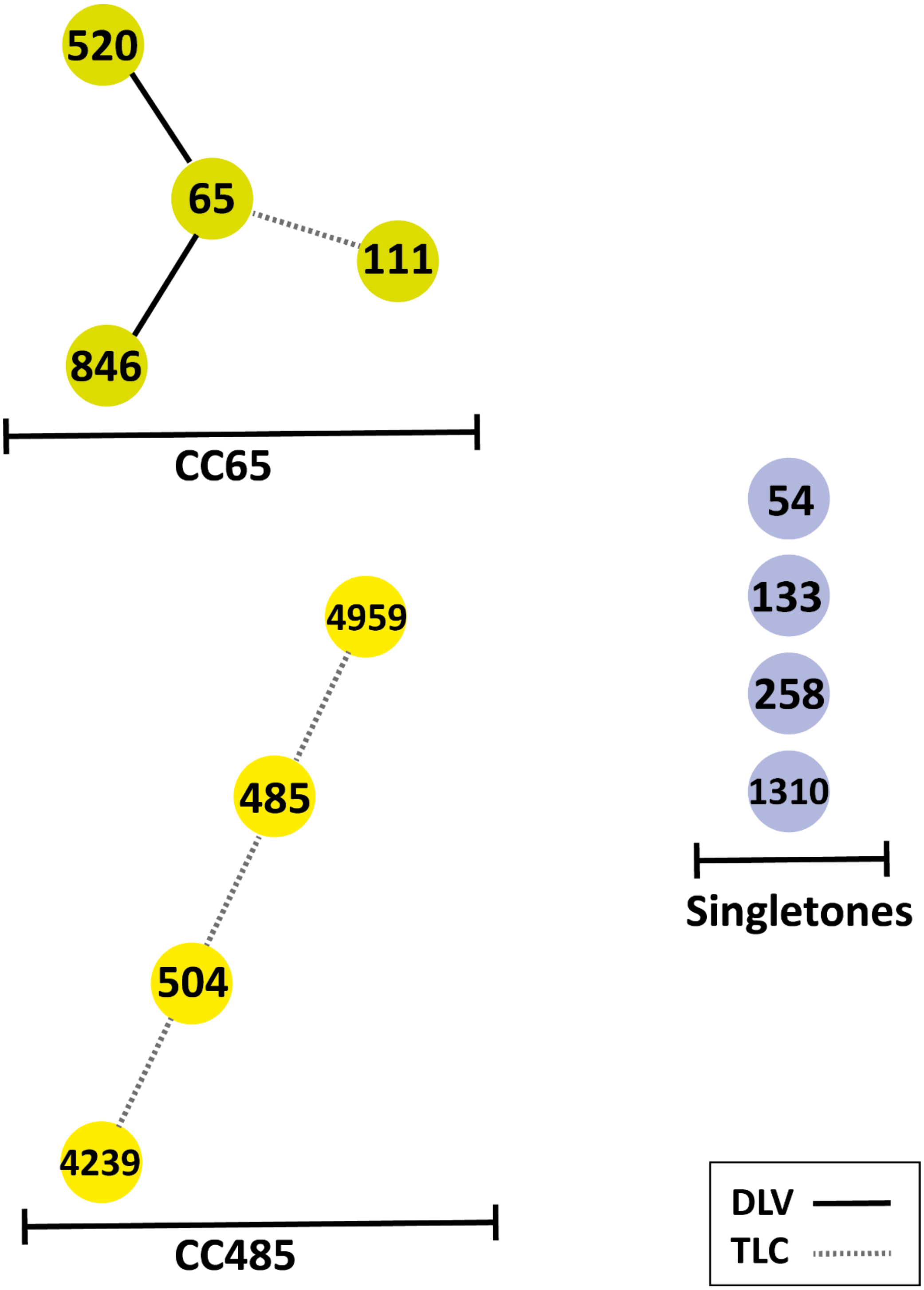

Figure S3. goeBURST analyses of KPC-*Kp* STs. Clonal complexes are represented in different colors, singletons in purple. STs with double locus (DLV) and triple locus variants (TLV) are joined by continues and dashed lines, respectively.

Table S3. Resistome of KPC-*Kp* clones and KPC-Kv isolate

Klebsiella specie	Isolate (ST)	Contig	DNA molecules	Length (bp)	Circular	Inc group	Resistance genes
K. pneumoniae	33Kpn9 (846)	33Kpn9	Chromosome	5,464,147	Yes	-	*fosA* and *oqxAB*
		p33Kpn9-KPC	Plasmid	132,884	Yes	IncFII(K)	*bla*$_{CTX-M}$, *bla*$_{OXA-1}$, *bla*$_{KPC-2}$, *aac(6 ')-Ib-cr*, *drfA14*, *tetA,* and *catB3*
		p33Kpn9-2	Plasmid	124,915	Yes	IncFIB(K)	None
K. pneumoniae	33Kpn12 ST504)	33Kpn12	Chromosome	5,285,656	Yes	-	*fosA* and *oqxAB*
		p33Kpn12-1	Plasmid	116,955	Yes	IncN	*bla*$_{TEM-1}$, *bla*$_{CTX-M-15}$, *aadA16*, *aph(6)-Id*, *aac(6 ')-Ib-cr*, *drfA27*, *sul1*, *sul2* and *arr3*
		p33Kpn12-KPC	Plasmid	15,946	Yes	ColRNAI	*bla*$_{TEM-1}$ and *bla*$_{KPC-2}$
K. pneumoniae	33Kpn22(ST258)	33Kpn22	Chromosome	5,501,536	Yes	-	*bla*$_{KPC-3}$, *fosA* and *oqxAB*
		p33Kpn22-1	Plasmid	196,777	Yes	IncF	*catA1*
		p33Kpn22-1	Plasmid	174,177	Yes	-	None
		p33Kpn22-1	Plasmid	107,862	Yes	IncFII(Yp)	*aac(6')-Ib* (two copies) and *aac(6')-Ib-cr* (two copies)
		p33Kpn22-KPC	Plasmid	74,766	Yes	(IncI2 (Δ)	*bla*$_{TEM-1A}$ and *bla*$_{KPC-3}$
		p33Kpn22-1	Plasmid	47,425	Yes	IncR	*aac(3)-IV*, *aac(6')-Ib*, *aadA1*, *aadA2b*, *aph(4)-Ia*, *cmlA1*, *aac(6')-Ib-cr* and *sul3*
K. variicola	33Kva16 (ST182)	33Kva16-1	Chromosome	2,411,593	No	-	*oqxAB*, *fosA*
		33Kva16-2	Chromosome	1,313,960	No	-	None
		33Kva16-3	Chromosome	428,564	No	-	*bla*$_{LEN-16}$
		33Kva16-4	Chromosome	400,815	No	-	None
		33Kva16-5	Chromosome	375,057	No	-	None
		33Kva16-6	Chromosome	330,604	No	-	None
		33Kva16-7	Chromosome	155,149	No	-	None
		p33Kva16-1	Plasmid	122,204	Yes	IncFIB(K)	*bla*$_{KPC-2,}$ *aadA16*, *aac(6 ')-Ib-cr*, *qnrB6*, *sul1*, *drfA27*, and *arr3*
		p33Kva16-KPC	Plasmid	57,529	Yes	IncN	None

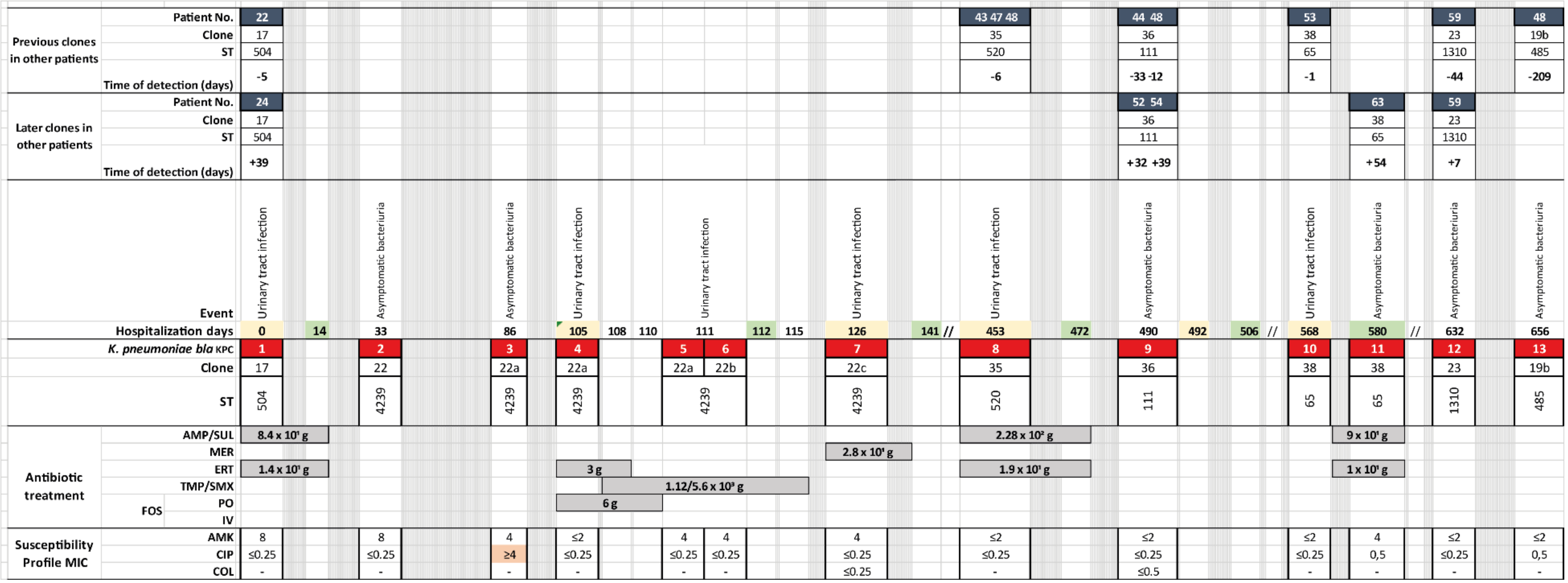

Figure S4. Analysis of the circulation dynamics of KPC-Kp clones of patient number 23 during antibiotic treatment during the hospital stay in 2015 and 2016. The figure illustrates the timeline, highlighting in yellow each admission of the patient to the institution and in green the discharges, with a description of the events associated with the KPC -*Kp* isolates, which are colored red, with their respective MLST. Regarding the susceptibility profile, only the antibiotics to which the isolates were susceptible were illustrated at the bottom, with the minimum inhibitory concentrations (MIC) in white boxes (CLSI cutoffs). The antibiotic and duration (in days) of treatment, with the cumulative dose in grams are described in gray boxes. Finally, the time connection with other patients (blue boxes) who presented the same clone and the number of days of difference are shown in the top.

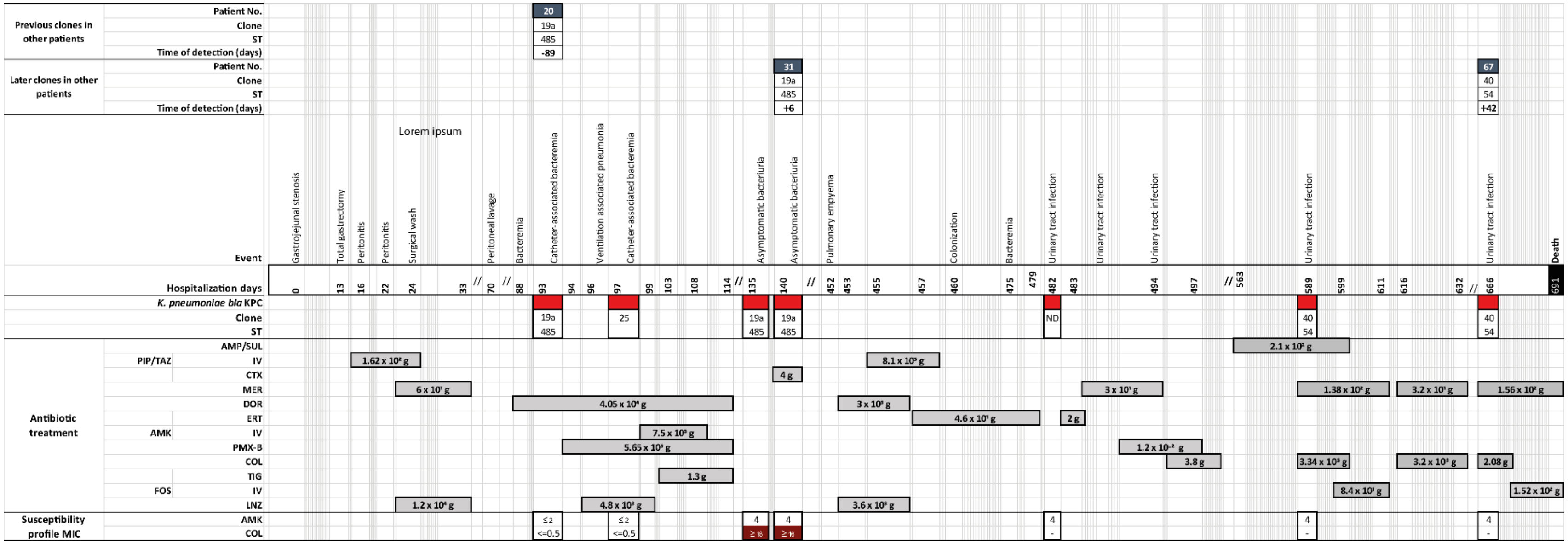

Figure S5. Analysis of the circulation dynamics of KPC-Kp clones of patient number 27 during antibiotic treatment in the hospital stay in 2015 and 2016. The graph illustrates to scale the timeline from the admission of the patient to the institution (day 0) until death (day 691), with a description of the events associated with the KPC -*Kp* isolates, which are colored red, with their respective MLST. Regarding the susceptibility profile, only the antibiotics to which the isolates were susceptible were illustrated at the bottom, with the minimum inhibitory concentrations (MIC) in white boxes (CLSI cutoffs). The antibiotic and duration (in days) of treatment, with the cumulative dose in grams are described in gray boxes. Finally, the time connection with other patients (blue boxes) who presented the same clone and the number of days of difference are shown in the top.

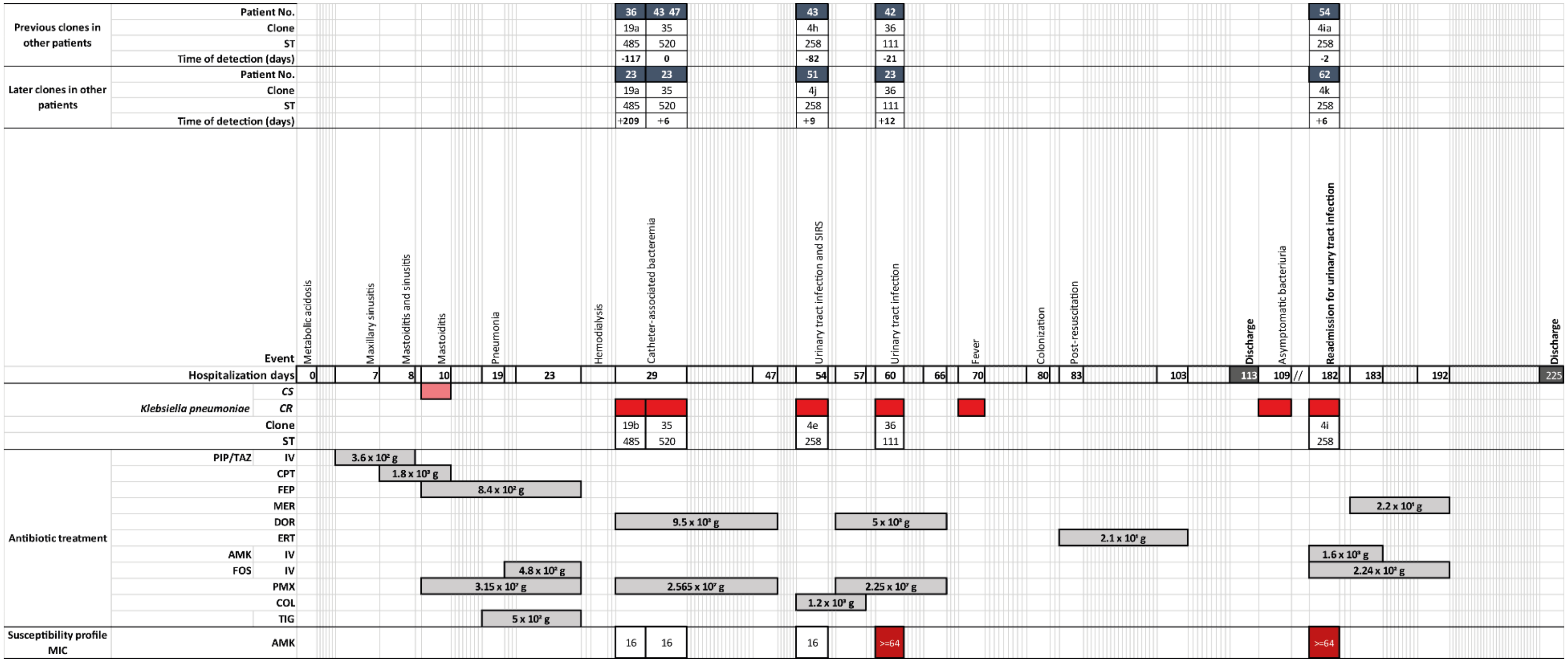

Figure S6. Analysis of the circulation dynamics of KPC-Kp clones of patient number 48 during antibiotic treatment in the hospital stay in 2016. The graph illustrates to scale the timeline, highlight admissions of the patient to the institution and discharges, with a description of the events associated with the KPC -*Kp* isolates, which are colored red, with their respective MLST. Regarding the susceptibility profile, only the antibiotics to which the isolates were susceptible were illustrated at the bottom, with the minimum inhibitory concentrations (MIC) in white boxes (CLSI cutoffs). The antibiotic and duration (in days) of treatment, with the cumulative dose in grams are described in gray boxes. Finally, the time connection with other patients (blue boxes) who presented the same clone and the number of days of difference are shown in the top.

Table S4. Comparison of the clinical characteristics of the patients who had ST258 isolates

Variable		Clone 4 (ST258)	Others clones	p value
Average age		69.5	52	0.03
Gender	Male	10	27	0.9
	Female	6	26	
Average stay		110	57	0.624
ICU admission	Yes	9	35	0.47
	No	7	18	
Hospitalization cause	Medical	10	23	0.18
	Surgical	6	30	
Charlson index		5.13	4.4	0.43
Outcome	Dead	2	10	0.55
	Alive	14	43	
Average APACHE		14.5	14.07	0.48
Average SOFA		7.14	5.19	0.32
Clinically significant event	Yes	23	61	0.1
	No	9	47	

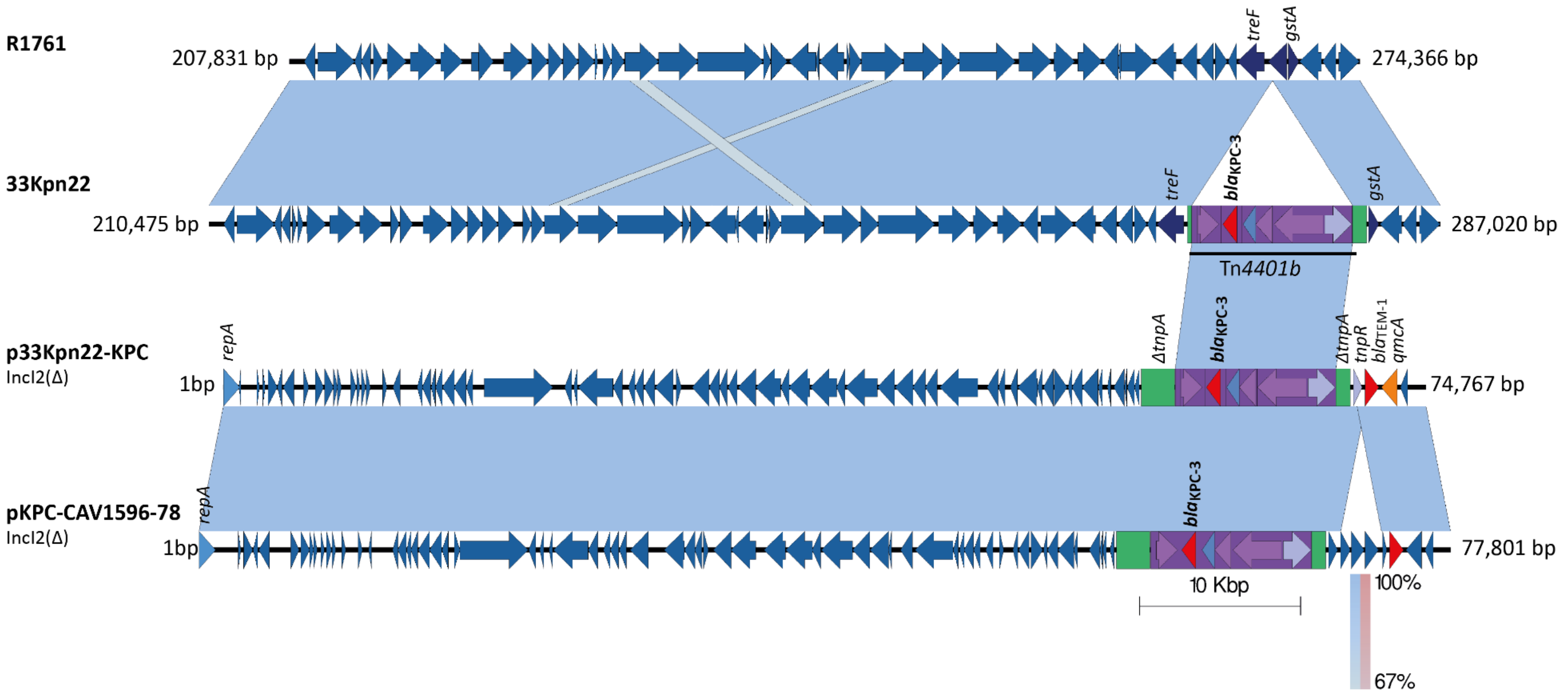

Figure S7. Insertion of the Tn*4401b* of the IncI2 (Δ) plasmid into the chromosome of *Klebsiella pneumoniae* 33Kpn22 isolate. The shaded area between the sequences delimits the regions with ≥67% alignment identity. Blue arrows indicate open reading frames; Coding regions for transposases and resolvases are shown in purple; Resistance genes in red; purple rectangles depict the Tn*4401b* transposons.
